# Robust genomic copy number predictor of pan cancer metastasis

**DOI:** 10.18632/genesandcancer.165

**Published:** 2018-01

**Authors:** Alexander Pearlman, Kinnari Upadhyay, Kim Cole, John Loke, Katherine Sun, Susan Fineberg, Stephen J. Freedland, Yongzhao Shao, Harry Ostrer

**Affiliations:** ^1^ Department of Pathology, Albert Einstein College of Medicine, Bronx, NY, USA; ^2^ Department of Pathology, NYU School of Medicine, New York, NY, USA; ^3^ Department of Surgery (Urology), Center for Integrated Research for Cancer and Lifestyle, Cedars-Sinai, Los Angeles, CA, USA and the Durham VA Medical Center, Durham, NC, USA; ^4^ Division of Biostatistics, NYU School of Medicine, New York, NY, USA

**Keywords:** Metastasis, cancer, genomic

## Abstract

Copy number alterations(CNAs) are the most common genetic changes observed in many cancers, reflecting the innate chromosomal instability of this disorder. Yet, how these alterations affect gene function to promote metastases across different tumor types has not been established. In this study, we developed a pan-cancer metastasis potential score (panMPS) based on observed CNAs. panMPS predicts metastasis and metastasis-free survival in cohorts of patients with prostate cancer, triple negative breast cancer and lung adenocarcinoma, and overall survival in the Metabric breast cancer cohort and three cohorts from The Cancer Genome Atlas (TCGA), including prostate, breast and lung adenocarcinoma. These CNAs are present in cell lines of metastatic tumors from eight different origins, reflected by an elevated panMPS for all cell lines. Many copy number alterations involve large chromosomal segments that encompass multiple genes (“clumps”). We show that harnessing this structural information to select only one gene per clump captures the contributions of other genes within the clump, resulting in a robust predictor of metastasis outcome. These sets of selected genes are distinct from cancer drivers that undergo mutation, and in fact, metastasis-related functions have been published for over half of them.

## INTRODUCTION

Tumor metastasis to distant sites accounts for 90% of solid tumor cancer deaths [1]. The frequency with which metastasis occurs varies by tumor type, and even within a tumor type the time between diagnosis to metastasis can be quite variable. Nonetheless, many of the steps involved in the development of metastasis are shared across tumor types, including detachment from the matrix of origin and evasion of apoptosis, invasion beyond the site of origin, and colonization of distant sites. These steps are genetically encoded [2]. Metastasis-promoting genes that alter cellular functions in cell lines and in animal models have been identified [1-3].

CNAs are the genetic changes most commonly observed in human cancers, reflecting the innate chromosomal instability of many tumors [2]. On average, one-third of a cancer genome demonstrates CNAs with roughly equal distributions of copy number gains and losses [6]. CNAs are accentuated when mutations occur in stability genes that affect DNA repair, mitotic recombination or chromosomal segregation [2]. Analysis of (CNAs) has proven useful to identify markers that are associated with metastasis within specific primary tumor types [4, 5]. A study from our laboratory showed that despite the high frequency of these CNAs throughout the genome, 366 genes within these regions were commonly altered with similar amplification and deletion patterns in prostate cancer metastases and primary tumors that progress to metastases [4]. Sixty-five percent of the genes (241 of 366) were structured on the genome as contiguous gene clumps comprising two through thirteen genes per clump with a total of 69 clumps. The remaining 35% of the genes (125 of 366) were observed as singletons.

Knowledge of these genes and their CNAs could provide clinical utility for predicting aggressive disease requiring treatment *versus* indolent disease that could be actively monitored. To make such predictions, we developed a metastatic potential score (MPS) that was based on the weighted frequency of specific CNA pattern in the 366 genes observed in prostate cancer metastases [4]. This frequency of the CNA pattern in metastasis-prone *versus* indolent tumors provided a basis for calculating Zgenes scores, a measure of the contribution for the specific genes that included a penalty when the CNA went in the opposite CNA direction (amplification or deletion of chromosomal region). The MPS score represents the sum of Zgenes scores, divided by the number of genes being summed. When applied to a cohort of 60 primary prostate tumors, of which 13 developed metastases, MPS accurately predicted in 80% of cases for the endpoint of metastasis-free survival [4]. Given that 366 genes identified within CNAs are linked with metastases in general, and not limited to prostate cancer, it is possible that these CNAs may be drivers of metastasis in other primary cancers and therefore represent a pan-cancer metastasis signal.

In this study, we assessed the prevalence of these CNAs among large numbers of primary prostate cancers, triple negative breast cancers, other breast cancers and lung adenocarcinomas with known outcome. We used a subset of the CNA genes to develop a predictive pan-cancer metastatic potential score (panMPS), because the four cohorts were assayed on different array platforms that represented different CNA genes. The panMPS was derived by using 295 of the 366 CNA genes that overlapped across all array platforms. Although 71 CNA genes were not represented in the panMPS, most of these were located in multi-gene clumps, thereby capturing the content of 67 of the 69 clumps, with no loss in the predictive accuracy for the panMPS relative to the MPS using 366 genes (Supplementary Table 1). We also observed high frequencies of these alterations in metastatic cell lines for tumors of eight different origins.

## RESULTS

### panMPS predicts risk of metastasis in prostate and triple negative breast cancers and lung adenocarcinoma

**Figure 1:**
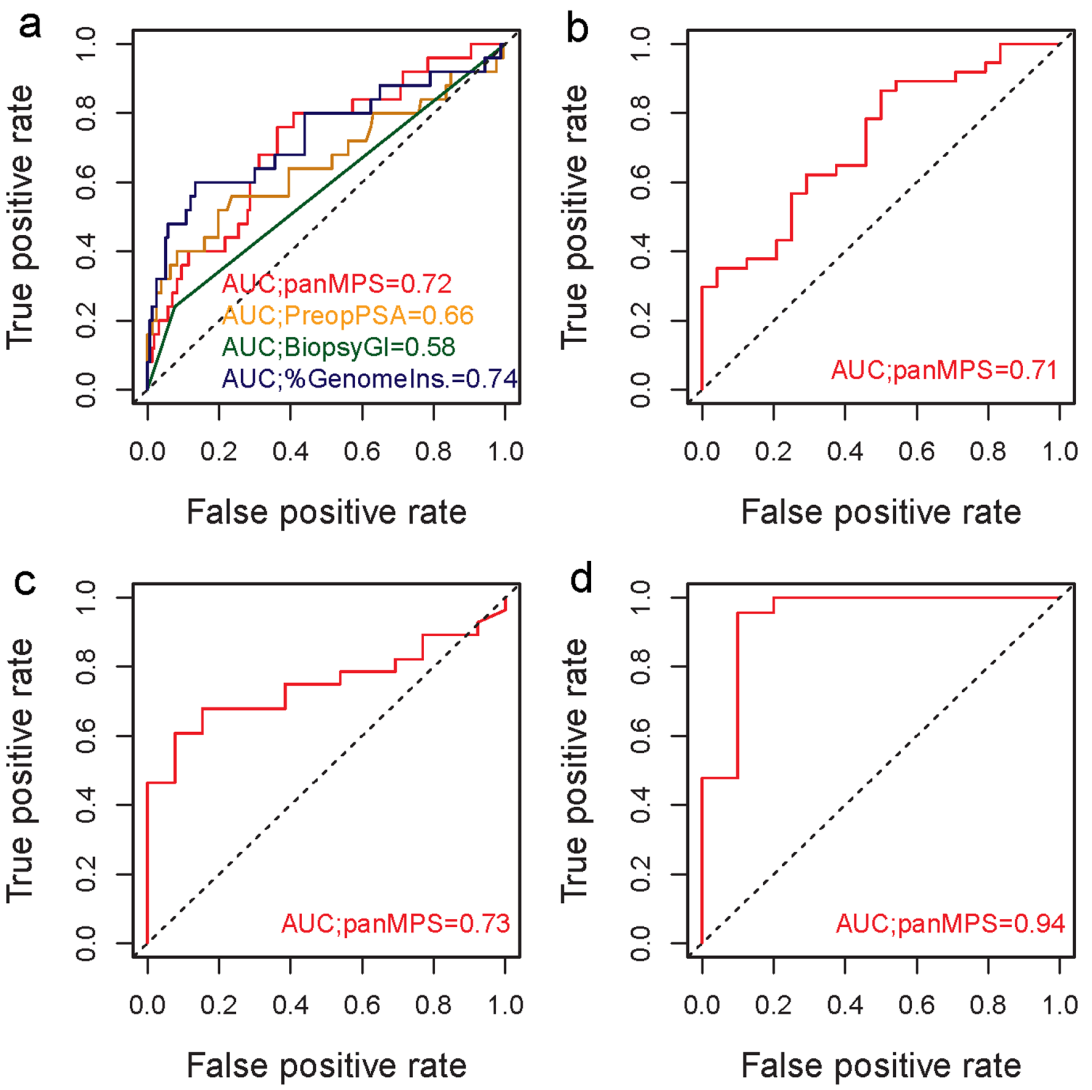
Receiver operating characteristic curves estimate the accuracy of the panMPS for predicting metastatic outcome for prostate cancer (**a.** MSK cohort *n* = 182, **b.** Duke cohort *n* = 61), triple negative breast cancer (**c.** Montefiore cohort *n* = 41) and lung adenocarcinoma (**d.** MSKCC cohort *n* = 33). Y axis indicates true positive rate and X indicates false positive rate. For prostate cancer, panMPS was predictive of mPT and iPT status in both the MSK and Duke cohorts. In addition, preoperative PSA, biopsy Gleason score, and percent genomic instability were predictive of mPT and iPT status in the MSK cohort, only. The AUC is indicated for each curve.

The validity of panMPS as a predictor of metastasis outcome was tested in studies of primary tumors, including prostate cancer, triple negative breast cancer and lung adenocarcinoma. For the outcome of prostate cancer metastasis, two cohorts were analyzed - one from Memorial Sloan Kettering Cancer Center (MSK; *n* = 182) and the other from Duke University (Duke; *n* = 61). Univariate logistic regression of panMPS resulted in significant odds ratios (OR) and areas under receiver-operator curves (AUCs) for the MSK (OR = 6.01, AUC = 0.71, *p* = 0.001) and the Duke (OR = 11.39, AUC = 0.72, *p* = 0.004) cohorts (Table 1 and Figure 1). Pre-operative PSA and pathology stage improved the AUC in logistic regression analysis of the MSK cohort, but did not lead to improvement in the Duke cohort due to matching between metastasis-prone primary tumors (mPTs) and indolent primary tumors (iPTs) for age, race, pathological stage, margin status, Gleason score, and surgery year, (Supplementary Table 2). Univariate logistic regression analysis of percent genomic instability in the MSK cohort, generated OR = 1.17, AUC = 0.74, *p* = 1.4X10^-5^ as reported previously (7); however, this predictor did not reach statistical significance in the Duke cohort (OR = 1.04, AUC = 0.80, *p* = 0.12; Supplementary Table 2). This result indicates that panMPS predicts prostate cancer metastasis and percent genomic instability, while useful in the MSK cohort, was not a strong independent predictor of metastasis in the Duke cohort.

**Table 1:** Univariate logistic regression model of panMPS predicts progression to metastasis for cancers

Cohort	MSK prostate cancer (*n* = 182, mPT = 25, iPT = 157)				Duke prostate cancer (*n* = 61, mPT = 37, iPT = 24)			
Variable	Odds Ratio	*P*	95% CI	AUC	Odds Ratio	*P*	95% CI	AUC
panMPS	6.01	0.001	2.21 to 17.89	0.71	11.39	0.004	2.39 to 70.36	0.72

Cohort	Montefiore triple negative breast cancer (*n* = 41, mBC = 28, iBC = 13)				MSK lung adenocarcinoma (*n* = 33, mLA = 23, iLA = 10)			
Variable	Odds Ratio	*P*	95% CI	AUC	Odds Ratio	*P*	95% CI	AUC
panMPS	44.74	0.02	2.91 to 1927.9	0.75	3.45×10 ^3^	0.006	41.5 to 1.26×10^7^	0.94

Similar analyses were performed on 41 samples from a Montefiore cohort of triple negative breast cancer metastasis. Univariate logistic regression of panMPS resulted in OR = 44.74, AUC = 0.75 and *p* = 0.02 (Table 1 and Figure 1). Percent genomic instability was not an independent predictor of metastasis (data not shown). Because matching had been performed for the triple negative breast cancers, stage was also not a predictor of metastasis outcome.

For the outcome of lung adenocarcinoma metastasis, univariate logistic regression of panMPS resulted in a significant AUC for the MSKCC cohort (OR = 3.45X10^3^, AUC = 0.94, *p* = 0.006; Table 1 and Figure 1). Because advanced stage cases for more survival and early-stage cases with less survival were selected, stage was not a valid predictor of metastasis.

### panMPS predicts metastasis-free survival (MFS) for prostate cancer, triple negative breast cancer and lung adenocarcinoma

As a univariate predictor through a Cox model analysis of MFS, panMPS was associated with prostate cancer metastasis-free survival in both the MSK (HR = 5.4, *p* = 0.0003, concordance index = 0.74) and Duke (HR = 3.4, *p* = 0.03, concordance index = 0.62) cohorts (Table 2). In univariate Cox analysis of the MSK cohort, percent genomic instability was associated with metastasis-free survival (HR = 1.11, *p* = 3.3X10^-7^, concordance index = 0.67), as previously reported for this cohort [7]; however, this variable did not reach statistical significance in the Duke cohort. Combining biopsy and pathological Gleason scores, preoperative PSA or pathological stage with panMPS predicted metastasis-free survival in Cox analysis of the MSK cohort, but not in the Duke cohort (Supplementary Table 3).

**Table 2:** Univariate Cox proportional hazards model of panMPS predicts metastasis-free survival for cancers

Cohort	MSK prostate cancer (*n* = 222, mPT = 25, iPT = 197)				Duke prostate cancer (*n* = 76, mPT = 37, iPT = 39)			
Variable	Hazard Ratio	95% CI	Conc- indx	*P*	Hazard Ratio	95% CI	Conc- indx	*P*
panMPS	5.42	2.18 to 13.49	0.74	0.0003	3.4	1.15 to 10.12	0.62	0.03

Cohort	Montefiore triple negative breast cancer (*n* = 41, mBC = 28, iBC = 13)				MSK lung adenocarcinoma (*n* = 33, mLA = 23, iLA = 10)			
Variable	Hazard Ratio	95% CI	Conc- indx	*P*	Hazard Ratio	95% CI	Conc- indx	*P*
panMPS	4.1	1.03 to 16.04	0.6	0.05	6.57	1.31 to 33.04	0.67	0.02

As a univariate predictor in a Cox model, panMPS was associated with triple negative breast cancer metastasis-free survival in the Montefiore cohort (HR = 4.1, *p* = 0.05, concordance index = 0.60; Table 2). Stage was also an independent predictor (HR = 3.2, *p* = 0.03), whereas percent genomic instability was not. As a Cox model univariate predictor, panMPS was associated with lung adenocarcinoma metastasis-free survival in the MSKCC cohort (HR = 6.6, *p* = 0.02, concordance index = 0.67; Table 2). As mentioned above, stage was not a valid a predictor due to matching.

### panMPS is associated with overall survival in breast cancer, prostate cancer and lung adenocarcinoma

**Figure 2:**
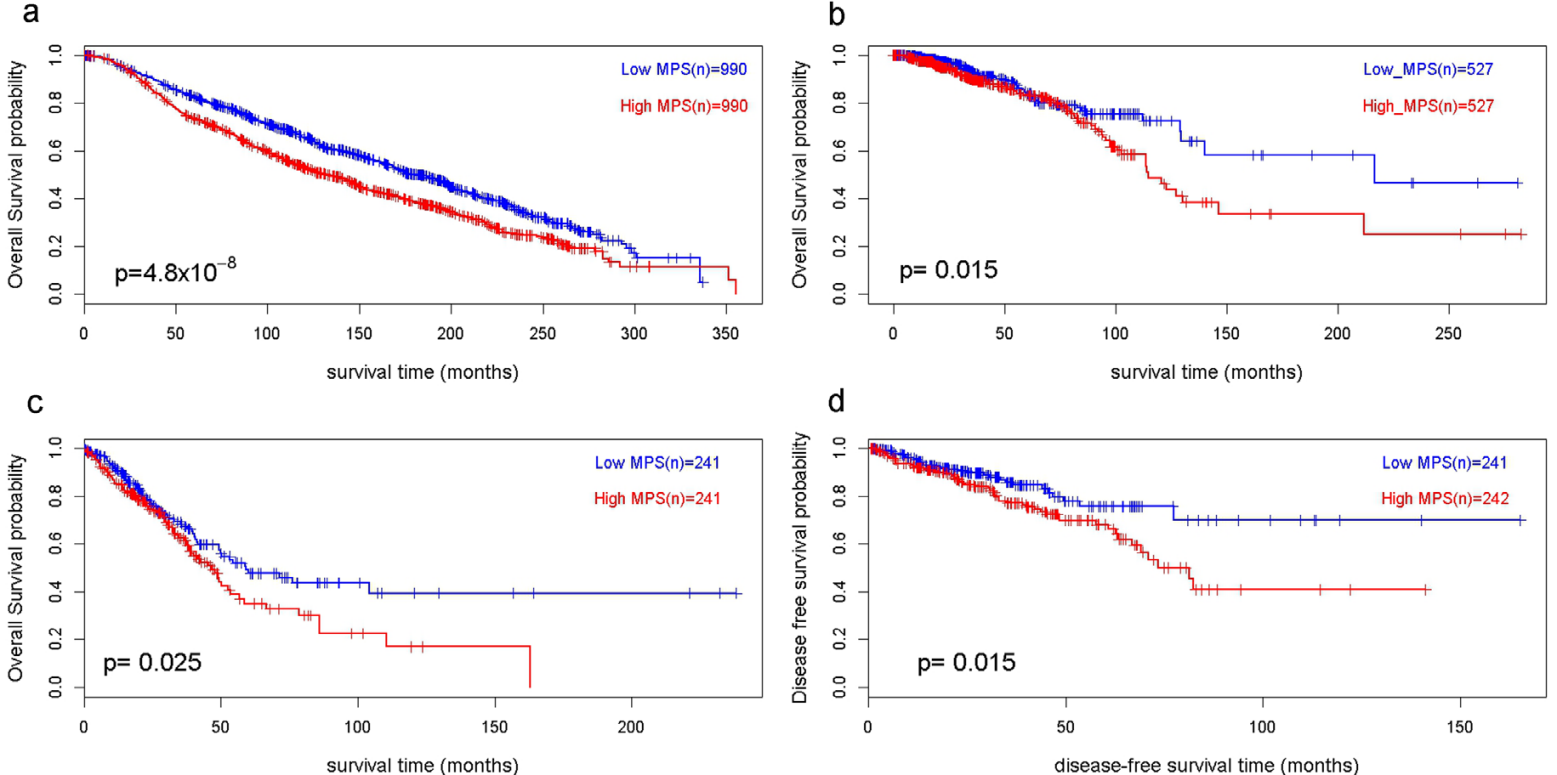
Kaplan Meier analysis shows that MPS is associated with overall survival **a.** Metabric breast cancer (*n*= 1980); **b.** TCGA breast cancer (*n* = 1054); **c.** TCGA prostate cancer (*n* = 482); **d.** TCGA lung adenocarcinoma (*n* = 483). Y-axis indicates overall survival probability and X axis indicates survival time. p-value calculated by log-rank test.

Data about CNAs in primary cancers and their survival outcomes are available for a variety of cancer types from publically available datasets, including The Cancer Genome Atlas (TCGA) [36, 37] and Metabric [38]. To examine general utility as a predictor of survival outcome, Kaplan Meier analysis of panMPS was applied to the TGCA prostate cancer, breast cancer, and lung adenocarcinoma cohorts and the Metabric breast cancer cohort. panMPS (median cut point) was observed to be significantly associated with overall survival in the Metabric breast cancer cohort (*n* = 1,980, *p* = 4.8×10^-8^) and in three TCGA cohorts (breast: *n* = 1054, *p* = 0.015, prostate: *n* = 483, *p* = 0.015, and lung adenocarcinoma: *n* = 482, *p* = 0.025; Figure 2), providing evidence that panMPS is a predictor not only of metastasis, but also survival. Metastasis-free survival data were not available for these cohorts.

### panMPS is elevated in many metastatic cancer cell lines of epithelial origin

To test applicability in other cancer types, genomic instability and panMPS were evaluated in a set of 133 cell lines of different tissue origins from the Cancer Cell Line Encyclopedia (CCLE). All cell lines were reported to be from metastatic tumors. The median number of protein coding genes demonstrating CNAs ranged from 2091 for lymphoma to 6805 for pancreatic carcinoma and 6916 for stomach carcinoma, thereby confirming the high frequency of CNAs in metastases (Supplementary Figure 1). By way of reference, the median number of genes demonstrating CNAs in a sample of clinical prostate cancer metastases was 3731. For metastatic cancer cell lines of epithelial origin, including breast, lung adenocarcinoma, pancreas and stomach, the frequency of CNAs was higher than those observed in prostate cancer metastases (*p* = 0.04, 0.002, 3X10^-4^, 0.005, respectively), whereas for metastatic cell lines of non-epithelial origin, including lymphoid tissue, melanoma, and lung small cell, the frequency of unstable genes was similar to that observed for prostate cancer metastases. Despite the higher frequencies of CNAs among metastatic cells lines of epithelial origin, the MPS of these cell lines, including breast, lung adenocarcinoma, pancreas, large intestine and stomach, was similar to that observed in prostate cancer metastasis. Cell lines of non-epithelial origin had either comparable (melanoma) or lower MPS (lymphoid - *p* = 8X10^-4^, lung small cell - *p* = 0.01) to those observed in clinical prostate cancer metastases. These findings extend the previous observation that the CNAs of cancer cell lines of a variety of origins display a specific CNA pattern [4], suggesting that panMPS might serve as a predictor of metastatic outcome across multiple cancer types.

### Annotation of MPS genes shows that they are more likely to have known roles in promoting metastasis or predicting metastatic outcomes than randomly selected genes

**Figure 3:**
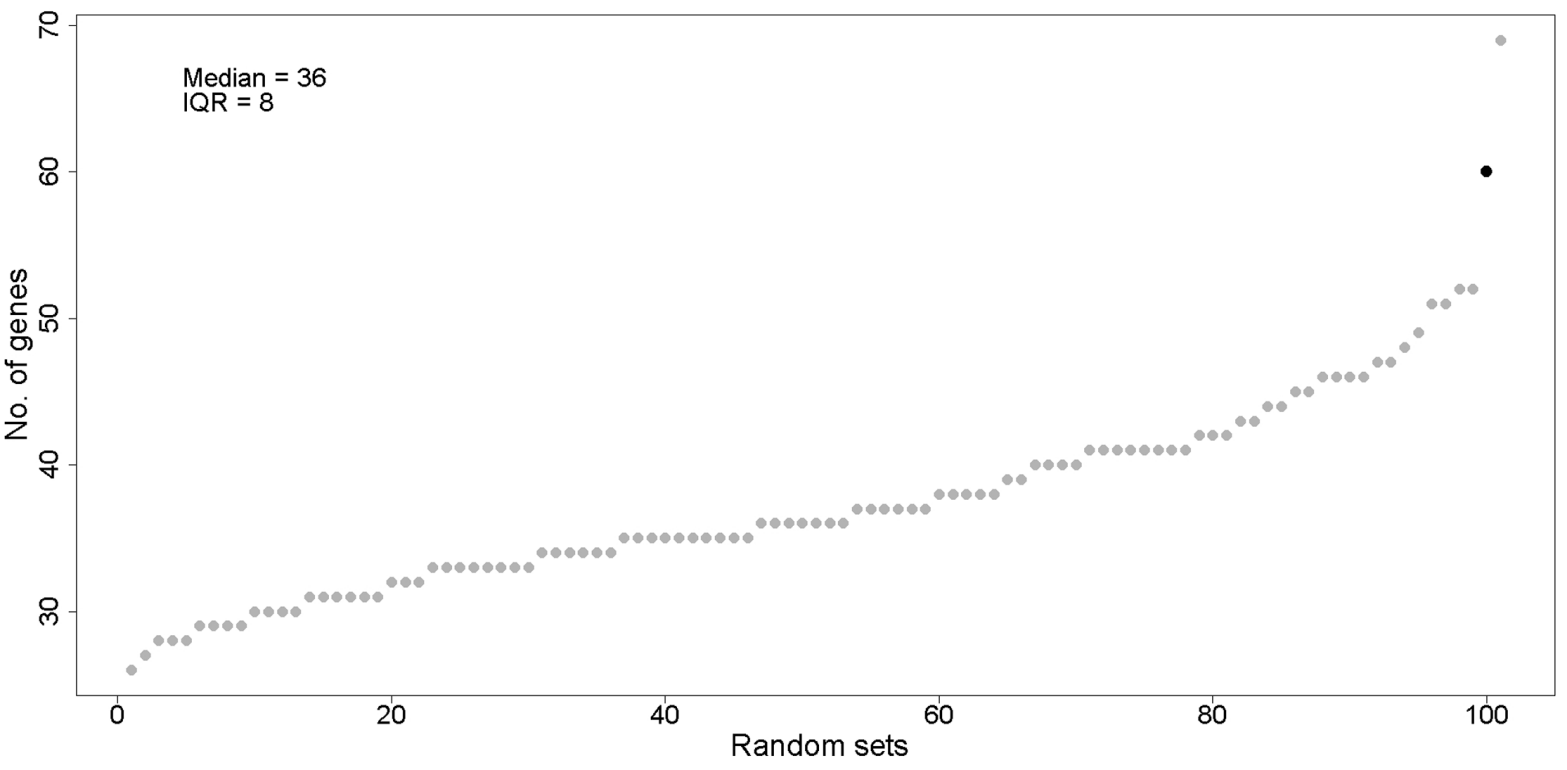
MPS genes show higher functional and biomarker annotations than random sets of genes Y-axis indicates number of genes found to have PubMed citations for metastasis functions for random sets of genes (grey) and MPS genes (black) and X-axis indicates each of 100 gene sets. There were 2 outliers that exceeded the upper fence, the MPS genes (*n* = 60) and one random set (*n* = 69).

Following guidelines for the functional interpretation of genes and their variants provided by the American College of Medical Genetics and Genomics [8], the Association for Molecular Pathology [9], and codified by the NIH-supported, Clinical Genome Resource [10], we annotated each of the 366 MPS genes for literature reports. Statistical tests were then performed, first to compare MPS genes to random gene sets for metastatic functions and the second of protein coding gene sets that have known associations with metastasis functions, such as invasion, motility and escape from apoptosis when detached from matrix of origin, and chemokine activity, and for biomarker genes predictive of metastasis outcome when their copy number or expression is altered (Supplementary Table 4). The frequency of literature reports of 366 genes was compared to the frequencies with which literature reports were observed for 100 random sets of 366 genes from the 18,638 protein coding genes that excluded overlapping MPS genes. Among the 366 genes, 60 were found in PubMed citations for the search terms related to metastasis functions and metastasis biomarkers, whereas the range for the random sets was 26 to 69 (Figure 3). Only a single random set was associated with a larger number of citations (*n* = 69) than for 366 MPS genes (*n* = 60), indicating that the panMPS represents a non-random gene set. In a second approach, all protein coding genes (*n* = 19004) in the genome were annotated for associations with metastasis (“Metastasis ID,” Table 3, Supplementary Table 5A), metastasis functions (“Metastasis functions,” Table 3, Supplementary Table 5B), as biomarkers that were predictive of metastasis (“Metastasis biomarkers,” Table 3, Supplementary Table 5C) or have chemokine activity (“Chemokine ID”, Table 3, Supplementary Table 5D). Of 2463 metastasis ID genes identified by literature annotation, 112 overlapped with MPS genes, indicating enrichment for this gene set (*p* = 2.42X10^-20^). Of 929 metastasis function genes, 40 overlapped with MPS genes (*p* = 1.18X10^-6^). Of the 687 metastasis biomarker genes, 28 overlapped with MPS genes (*p* = 0.0001). Of the 3126 chemokine genes, 65 overlapped with MPS genes (*p* = 0.04). Thus, MPS genes were enriched among gene sets with terms for metastasis function or metastasis biomarkers, and chemokine function in published studies.

**Table 3:** Hypergeometric analysis of MPS genes *versus* in silico gene sets for metastasis biomarker and metastasis function reviewed by cellular assays. Metastasis ID and chemokine ID terms in article title or abstract

Gene Set	Overlap	Gene set size	Overlap %	*P*
Metastasis biomarkers	28	687	4.08	0.0001
Metastasis function	40	929	4.31	1.18×10^-6^
Metastasis ID	112	2463	4.55	2.42×10^-20^
Chemokine ID	65	3126	2.08	0.04

### Elevated Z_genes_ scores vary for genes within a clump of contiguous amplified or deleted genes

**Figure 4:**
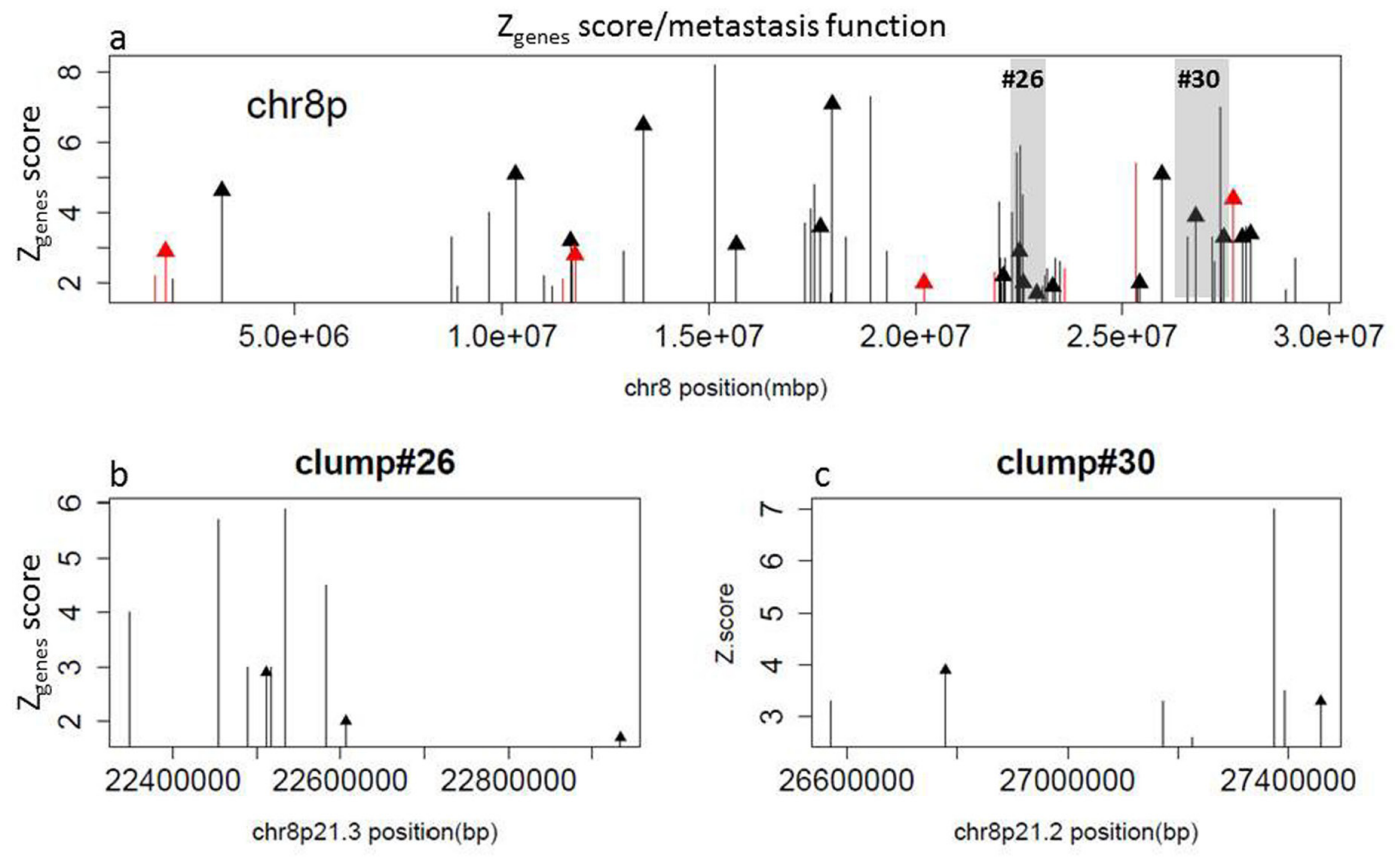
Chromosome 8p comprises 70 genes predictive of metastatic potential, including genes that occur in clumps (top panel) Each bar represents a gene as it is located along the chromosome (X-axis, base-pair number) whereas the height of the bar denotes a Zgenes score (Y-axis) that measures its ability to predict the metastatic potential of primary prostate cancer. Arrows on top of some of the bars indicate that the gene has been validated in prior metastasis studies as a biomarker or to have metastatic function. Clump region #26 (nine gene segment) and clump region #30 (seven gene segment) are highlighted in the top panel and zoomed in the bottom panels.

Genes that contribute to MPS can occur as singleton CNAs as well as in clumps that are distributed over 15 chromosomal arms (Supplementary Table 4). Genes within a clump (ranked by their relative contributions to MPS) are likely to include both drivers that are directly associated with metastasis function, and passengers that are associated with metastasis function by virtue of their proximity to metastasis driver genes. For example, a clump index 26 on chromosome 8p21.3 includes a region with nine contiguously deleted genes, PPP3CC, KIAA1967, BIN3, SORBS3, PDLIM2, RHOBTB2, SLC39A14, EGR3, and C8orf58 (Supplementary Table 4). In this clump, three of the 9 genes (EGR3, PDILMS, and RHOBTB2) overlapped with genes identified by different search terms (“metastasis ID”, ”metastasis functions” and “metastasis biomarkers”, suggesting that deletion of genes within this clump may promote distant metastases by different mechanisms. In addition to functional annotations, another way of gauging whether some of the MPS genes are metastasis drivers is to compare Zgenes scores within clumps [4]. Gene clumps vary by breakpoints in individual cancer genomes, and the CNAs of some genes in a clump will yield higher Zgenes scores by being overrepresented in a patient population, and in the specific pattern for metastasis, compared to cancer genomes that are not metastatic. As demonstrated on a frequently unstable region of chromosome 8p, the range of Zgenes scores within a clump varied from 1.7 to over 10. Within this region, there was no apparent pattern of decay from the highest Zgenes score gene to the lowest Zgenes score (Figure 4). Multiple genes within a clump had functional annotations, and were not necessarily those with the highest Zgenes score. Other unannotated MPS genes on chromosomes 8q and 16q with high Zgenes scores may also act as drivers of metastasis, but remain to be studied for functional roles (Supplementary Figures 2 and 3).

Genes such as CDH13, CDH8, CDH2, CTD8, COL19A1, YWHAG and ENOX1, do not belong to any clump. However, both the Zgenes scores and the annotations of these genes suggest that they may be drivers of metastasis (Supplementary Table 4). However, their functions may overlap, e.g., the cadherin genes, CDH13, CDH8, CDH2. Thus, there may be functional redundancy among MPS genes with several genes acting by the same molecular pathway. Yet, some of these genes have higher Zgenes scores suggesting that their contributions to metastasis are observed more frequently.

### High Z_genes_ score genes within clumps predict outcomes

**Figure 5:**
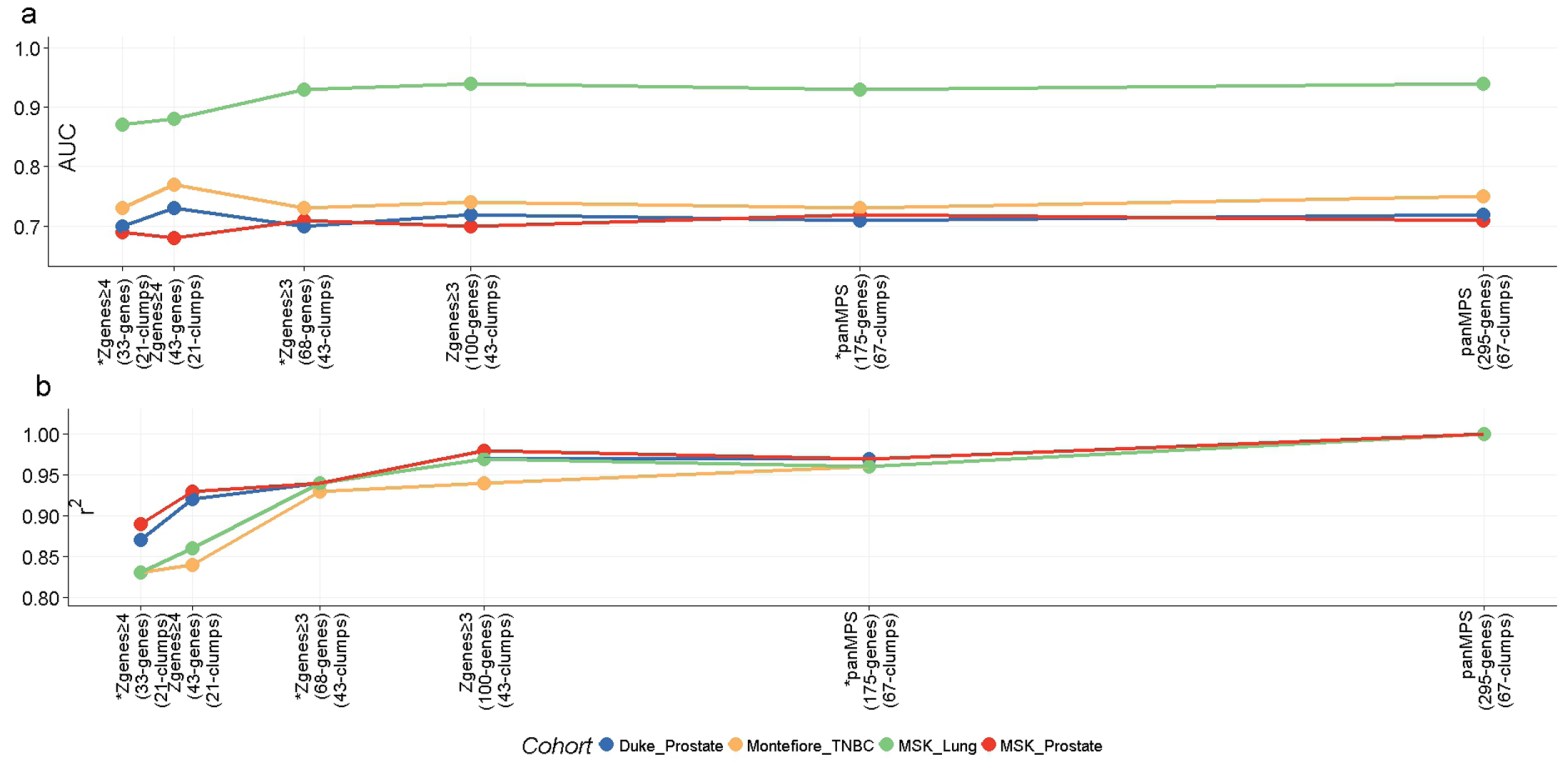
Small sets of high Z_genes_ score genes predict metastatic outcome and panMPS almost as well as all MPS genes for all four cohorts **a.** AUC (Y-axis) and **b.** r2 (Y-axis) estimate metastatic outcome and panMPS, respectively, for 295 genes and simplified versions, including fewer clumps of genes with high Zgenes scores. Numbers of genes and clumps are indicated for the different Zgenes scores (X-axis). * one gene per clump.

To test whether a reduced set of gene clumps could predict outcomes and produce similar values to those observed with panMPS, receiver operating characteristic - area under the curve (ROC-AUC) and linear regression r2 values were calculated for simplified MPS versions that included genes with Zgenes score ≥4 (21 clumps with 43 genes) and Zgenes score ≥3 (43 clumps with 100 genes) or the gene with the highest Zgenes score within a clump (Figure 5). The results were compared to panMPS which is calculated with all 295 genes estimating each MPS version's ability to accurately classify mPTs and iPTs (ROC-AUC) and the linearity of (r^2^). Both sets of gene clumps as well as the single genes with the highest Zgenes score had predicted ROC-AUC and r^2^ closely aligned to panMPS for all cohorts (Supplementary Tables 6A and 6B). For the CCLE cell lines, comparison was performed only for r2 and similar results were obtained (Supplementary Table 6C). This result indicated that there was a hierarchy of clumps with some clumps having higher Zgenes scores compared to others. The 21 clumps with Zgenes score ≥4 performed almost as well as the 43 clumps with Zgenes score ≥3, capturing almost all of the contribution of the clump to ROC-AUC and r^2^. Significantly, these result also show that a lead gene (highest Zgenes score) within a clump could capture almost all of the contribution of the clump to AUC and panMPS r2.

## DISCUSSION

CNAs are the result of chromosomal instability and are far more common than mutations in human cancers, including prostate, triple negative breast cancer and lung adenocarcinoma [2, 11]. CNAs may occur randomly across the genome or may be favored by repeated structural elements, including Alu or LINE sequences [12]. Amplifications or deletions of genes may occur repeatedly within the same regions of genomes in populations of cancer cells within a tumor [4, 13]. This observation of specific CNA pattern enrichment is the basis for calculating Zgenes scores for specific genes within CNAs. In turn, MPS represents the sum of Zgenes scores, divided by the number of genes being summed. CNA burden alone (i.e. the frequency of chromosomal instability) was not an accurate predictor of outcome in most cohorts because it did not consider specific pattern nor functional contributions by specific metastatic genes.

This study provides evidence that panMPS can be used as a predictor of metastasis and metastasis-free survival, not only in prostate cancer, as we have shown before [4], but also for triple negative breast cancer, other breast cancers, and lung adenocarcinoma and 133 CCLE metastasis cell lines of 8 different cancer origins. A panMPS was also able to predict overall survival in Metabric cohort of breast cancer and several large TCGA cohorts of prostate cancer, breast cancer and lung adenocarcinoma.

These observations fit a model of chromosomal rearrangements occurring in early tumorigenesis by punctuated bursts [14]. Metastasis is driven by selection for rearrangements that promote invasion, escape from apoptosis and growth at distant sites [1]. A study of mutated genes in multiple cancer types drew a similar conclusion that genes under positive selection, either in individual or multiple tumor sites, tend to display higher mutation frequencies above background [11]. However, large-scale targeted and whole genome sequence efforts have identified single nucleotide variants and short indels in a set of overlapping or related genes that account for carcinogenesis, but have not identified genes involved in metastasis [15].

These CNAs occur on a segmental basis with multiple genes within a segment or clump being amplified or deleted. Within a clump, one or more genes could be drivers of metastasis [11]. The drivers showed elevated Zgenes scores and were annotated in the literature as having metastatic functions, including invasion, motility, escape from apoptosis when detached from matrix of origin, and chemokine activity. Other genes with elevated Zgenes scores, but no annotations, may also represent drivers whose functions have not yet been identified. However, the remainder of the genes may be passengers that are carried along with the CNA events. Not all of the drivers are required for predicting risk of metastasis. Testing only genes with the highest Zgenes score within a clump may capture most, if not all of the metastatic risk, reflected by the panMPS. These genes with high Zgenes score may act as proxies for all of the genes within the clump.

Based on the hypergeometric analysis, the MPS genes indeed represent a subset of all metastatic genes, specifically those that can be readily identified by CNA analysis. Other metastatic genes would not be readily detected as they are not subject to CNA events and may need to be detected by other molecular methods, such as sequencing.

Having a test that would accurately predict across cancer-types which patients are likely to develop metastases would be extremely useful. For example, panMPS could improve the clinical management of men with prostate cancer. Men with early-stage disease and low-risk profiles would be candidates for active surveillance that might safely preserve quality of life by helping them to avoid erectile dysfunction and urinary incontinence that may occur in up to 50% of treated patients [17, 18]. Men with early-stage disease and high-risk profiles might benefit from aggressive treatment [19]. Men with higher-risk disease who underwent initial surgery might benefit from adjuvant radiation therapy [20]. Notably, the accuracy of combined panMPS and pre-operative PSA appears to be similar to the various RNA expression profile tests plus clinical predictors for use as a post-surgical tool (Supplementary Table 7). These tests, Genomic Prostate Score (GPS) [21, 22], Cell Cycle Progression Score (CCPS) [23], and Genomic Classifier (GC) [24-28], measure the altered expression of mostly non-overlapping sets of genes that have not been demonstrated to play a direct role with the biological events of prostate cancer progression and metastasis. As with panMPS, the accuracy of these tests was improved by the addition of clinical and pathological predictors, both as univariate predictors or as captured by the Cancer of the Prostate Risk Assessment (CAPRA-S) score [29, 30], and the Stephenson nomogram [31]. Although Oncotype DX and Prosigna are two RNA expression profile tests in common use for prognosis of breast cancer, their use is limited to estrogen receptor positive breast cancer [32, 33].

Analyzing panMPS genes in patient samples may be required to improve the accuracy of predicting metastasis- although the current study suggests that as few as 33 genes with high Zgenes score may be sufficient for many clinical applications.

The availability of a panMPS-based diagnostic tool may contribute to clinical care. Collectively, lung, breast and prostate cancer account for ~676,000 or 40% of newly diagnosed cancer cases and ~226,000 or 39% of cancer deaths in the United States each year [16]. Currently, there are no clinical tests in common use for prediction of outcomes in triple negative breast cancer or lung adenocarcinoma. Future studies will assess the accuracy of panMPS derived from surgical specimens and biopsies for predicting outcomes of these diseases.

## MATERIALS AND METHODS

### Predictive CNAs, MPS and panMPS

This study provides an in-depth analysis of a set of 366 genes found in CNAs that are predictive of prostate cancer metastasis. The contributions of these genes to MPS are reflected as Zgenes scores. These are calculated by assigning each probe on the array to a gene, provided it falls within 10,000 bp upstream or downstream of the transcription start or stop site.z = (X - μ) / σ as described previously [4]. The score for a gene, X, is subtracted by the mean, μ, of the background distribution of selection model scores and divided by the standard deviation, σ, of the background distribution of selection model scores. A conservative background distribution of selection model scores was calculated by sampling the top 5th percentile of amplified or deleted probes from all genes on the array with the same number of probes as the gene in question. The result is a Zgenes score for each gene in the genome that is represented on the array. Alternatively, the complete set of genomic CNAs was used to calculate percent genomic instability. The CNA methodology is assay platform-independent, but requires that genomic DNA signal intensities are measured within the regions of the metastasis signature. In this study, the analysis was conducted on primary data sets reported here utilizing the Affymetrix Oncoscan FFPE V3 array [34], and on previously generated data sets assayed on Agilent 240K and other arrays [7]. For comparison of cohorts from different platforms, the corresponding numbers of the MPS genes were reduced to include only those that overlapped (366 to 295 genes), representing the panMPS.

### Cohorts, tissue and sample processing

A prostate cancer radical prostatectomy cohort of 37 men that progressed to metastasis (mPTs) and 24 men that were free from biochemical recurrence (defined as PSA > 0.2 ng/ml, two values at 0.2 ng/ml or treatment for an elevated PSA) and metastases (iPTs) after at least five years of follow up was collected at Duke University (Duke cohort - Supplementary Table 7A). The Duke cohort had a case-control design that matched mPTs and iPTs for age, race, pathological stage, margin status, Gleason score, and surgery year. Tumor regions were microdissected, extracted for DNA, and assayed on the Oncoscan FFPE V3 array (Affymetrix Oncoscan Service, Santa Clara, California).

A second prostate cancer cohort, comprised of 25 mPTs along with 157 iPTs was collected at Memorial Sloan-Kettering Cancer Center (MSK cohort - Supplementary Table 7A). The collection, extraction and data generation for the second cohort has been described previously [7]. The MSK cohort represented a consecutive case-cohort design with non-recurrent, non-metastatic outcome samples making up a disproportionate number. Unlike the Duke samples, these samples were not matched for any criteria. The MSK cohort was comprised of fresh frozen radical prostatectomies. The Duke and MSK cohorts differed in their length of follow-up, clinical and pathologic attributes and biochemical recurrence and metastasis outcomes (SupplementaryTable 8A). The Duke cohort was collected for individuals with greater than five years follow-up since the majority of prostate cancers recur or metastasize within this timeframe. To achieve parity for prediction modeling and maximizing the metastasis informativeness of each patient, the MSK cohort was filtered for subjects that had at least five years of follow-up. Also, for both cohorts, metastasis negative subjects treated with radical prostatectomy and adjuvant radiation and/or hormonal therapy were excluded from analysis to provide a more homogeneous iPT group.

A triple negative breast cancer radical surgical cohort of 28 women that progressed to metastasis (mBCs) and 13 women that were free from local recurrence and metastasis (iBCs) after at least five years of follow up was collected at Montefiore Medical Center (Montefiore cohort - Supplementary Table 8B). The Montefiore cohort had a case-control design that matched mBCs and iBCs for age, race, pathological stage, margin status, and surgery year. The breast cancer tumor blocks from each patient were handled in a fashion similar to the prostate cancer tumor blocks. They were reviewed by a single pathologist and shown to be negative for expression of the estrogen receptor, progresterone receptor and HER2/NEU protein, as judged by immunohistochemistry. Tumor regions were microdissected, extracted for DNA, and assayed on the Oncoscan FFPE V2 array (Affymetrix Oncoscan Service, Santa Clara, California).

Tumor tissue from 199 primary lung adenocarcinomas was collected at the time of resection between 1996 and 2006 at MSKCC and analyzed for CNAs on Agilent 44K CGH arrays, as described previously [35]. From this cohort we selected all available early stage (1A,B and 2A,B) samples that progressed to mortality (mLA, *n* = 23) and late stage (3B and 4) samples that survived for more than one year after follow up (iLA, *n* = 10) (Supplementary Table 8C). This study was reviewed and approved by the Institutional Review Boards at Albert Einstein College of Medicine, New York University School of Medicine, and Duke University.

We downloaded the copy number alterations (CNAs) level 3 data from cBioPortal for cancer genomics (http://www.cbioportal.org/) for 3998 patients with three tumor types (Supplementary Table 9) [36, 37]. We selected Metabric and TCGA provisional study for breast invasive carcinoma, TCGA provisional study for lung adenocarcinoma and TCGA provisional study for prostate adenocarcinoma [38, 39]. We calculated panMPS score based on CNAs for these studies. Univariate Cox proportional hazards model was used to examine the association between MPS and survival. Overall survival was used as the endpoint.

### Cell lines

CNA data from 183 human cell lines of metastatic origin were available from the Cancer Cell Line Encyclopedia (CCLE). These cell lines included breast, lung adeno, pancreas, large intestine, lymphoid, melanoma, lung small cell and stomach cancers. The data were generated using the Affymetrix SNP 6.0 arrays, as described previously [6].

### MPS and panMPS

MPS was calculated based on genomic CNAs overlapping 366 genes with a higher score indicating a greater likelihood of metastasis, as described previously [4]. The pan cancer MPS or panMPS was derived from the MPS by using a subset of 295 genes from the MPS. Univariate and multivariate logistic regression and Cox proportional hazards survival models for prostate cancer were evaluated for panMPS, pre-surgery predictors (PSA, clinical stage, biopsy Gleason), demographic variables (age at diagnosis and race), and percent genomic instability, as described previously [7]. The logistic regression and Cox models were also tested for triple negative breast cancer and lung adenocarcinoma. AUC and concordance index were calculated for the logistic and Cox models, respectively.

### Functions of CNA genes in driving metastases

To gauge whether the recurrent CNA genes played a functional role in metastasis, we performed in-silico analysis by running three comprehensive queries with the RISmed package from R. First we performed a general PubMed citation query by searching for the 366 gene IDs and the terms “metastasis”, “metastases” or “metastatic” in the title or abstract of the publication (“metastasis IDs”). Next, we appended this query to capture metastasis functions by adding search terms, “apoptosis assay”, ”TUNEL”, ”Matrigel”, ”invasion assay”, ”wound healing assay”, ”migration assay”, ”MTT”, ”BrDU”, ”proliferation assay”, ”SiRNA” and “xenograft” (“metastasis functions”) or “chemokine” (“chemokine ID”). Then, we appended the title query to capture predictive biomarkers of metastasis by adding search terms, “Cox”, “Kaplan-Meier” and “hazard ratio” (“metastasis biomarkers”). The gene queries were manually curated and confirmed for accuracy by two reviewers. The annotation frequency was computed for each query type. To assess the significance of these annotations for the recurrent CNA genes compared to the remaining, non-overlapping 18,638 human protein coding genes an enrichment analysis based on the hypergeometric distribution was performed for the recurrent CNA genes *versus* all 19,004 protein coding genes annotated using the same query search terms to create expanded gene sets for metastasis ID, metastasis functions, metastasis biomarkers and chemokine ID.

### Reduction of complexity

To determine whether the genes with the highest Zgenes score among the clumps could predict outcomes as well as the full set of 295 genes for panMPS, we calculated AUC and r2 for simplified MPS versions by using genes with Zgenesscore ≥ 3, Zgenes score ≥ 4, or highest Zgenes score within a clump.

## SUPPLEMENTARY MATERIAL TABLES AND FIGURES



## References

[1] Nguyen DX, Massague J (2007). Genetic determinants of cancer metastasis. Nat Rev Genet.

[2] Vogelstein B, Kinzler KW (2004). Cancer genes and the pathways they control. Nat Med.

[3] Hunter KW (2004). Host genetics and tumour metastasis. Br J Cancer.

[4] Pearlman A, Campbell C, Brooks E, Genshaft A, Shajahan S, Ittman M, Bova GS, Melamed J, Holcomb I, Schneider RJ, Ostrer H (2012). Clustering-based method for developing a genomic copy number alteration signature for predicting the metastatic potential of prostate cancer. J Probab Stat.

[5] Taylor BS, Schultz N, Hieronymus H, Gopalan A, Xiao Y, Carver BS, Arora VK, Kaushik P, Cerami E, Reva B, Antipin Y, Mitsiades N, Landers T (2010). Integrative genomic profiling of human prostate cancer. Cancer Cell.

[6] Beroukhim R, Mermel CH, Porter D, Wei G, Raychaudhuri S, Donovan J, Barretina J, Boehm JS, Dobson J, Urashima M, Mc Henry KT, Pinchback RM, Ligon AH (2010). The landscape of somatic copy-number alteration across human cancers. Nature.

[7] Hieronymus H, Schultz N, Gopalan A, Carver BS, Chang MT, Xiao Y, Heguy A, Huberman K, Bernstein M, Assel M, Murali R, Vickers A (2014). Copy number alteration burden predicts prostate cancer relapse. Proc Natl Acad Sci U S A.

[8] Richards S, Aziz N, Bale S, Bick D, Das S, Gastier-Foster J, Grody WW, Hegde M, Lyon E, Spector E, Voelkerding K, Rehm HL, Committee ALQA (2015). Standards and guidelines for the interpretation of sequence variants: a joint consensus recommendation of the American College of Medical Genetics and Genomics and the Association for Molecular Pathology. Genet Med.

[9] Rehm HL, Berg JS, Brooks LD, Bustamante CD, Evans JP, Landrum MJ, Ledbetter DH, Maglott DR, Martin CL, Nussbaum RL, Plon SE, Ramos EM, Sherry ST (2015). ClinGen. ClinGen--the Clinical Genome Resource. N Engl J Med.

[10] Strande NT, Riggs ER, Buchanan AH, Ceyhan-Birsoy O, DiStefano M, Dwight SS, Goldstein J, Ghosh R, Seifert BA, Sneddon TP, Wright MW, Milko LV, Cherry JM (2017). Evaluating the clinical validity of gene-disease associations: An evidence-based framework developed by the Clinical Genome Resource. Am J Hum Genet.

[11] Kandoth C, McLellan MD, Vandin F, Ye K, Niu B, Lu C, Xie M, Zhang Q, McMichael JF, Wyczalkowski MA, Leiserson MD, Miller CA, Welch JS (2013). Mutational landscape and significance across 12 major cancer types. Nature.

[12] Aguilera A, Garcia-Muse T (2013). Causes of genome instability. Annu Rev Genet.

[13] Shah SP, Roth A, Goya R, Oloumi A, Ha G, Zhao Y, Turashvili G, Ding J, Tse K, Haffari G, Bashashati A, Prentice LM, Khattra J (2012). The clonal and mutational evolution spectrum of primary triple-negative breast cancers. Nature.

[14] Gao R, Davis A, McDonald TO, Sei E, Shi X, Wang Y, Tsai PC, Casasent A, Waters J, Zhang H, Meric-Bernstam F, Michor F, Navin NE (2016). Punctuated copy number evolution and clonal stasis in triple-negative breast cancer. Nat Genet.

[15] Kan Z, Jaiswal BS, Stinson J, Janakiraman V, Bhatt D, Stern HM, Yue P, Haverty PM, Bourgon R, Zheng J, Moorhead M, Chaudhuri S, Tomsho LP (2010). Diverse somatic mutation patterns and pathway alterations in human cancers. Nature.

[16] Siegel RL, Miller KD, Jemal A (2015). Cancer statistics, 2015. CA Cancer J Clin.

[17] Cooperberg MR, Broering JM, Carroll PR (2009). Risk assessment for prostate cancer metastasis and mortality at the time of diagnosis. J Natl Cancer Inst.

[18] Paris PL, Weinberg V, Albo G, Roy R, Burke C, Simko J, Carroll P, Collins C (2010). A group of genome-based biomarkers that add to a Kattan nomogram for predicting progression in men with high-risk prostate cancer. Clinical Cancer Research: an official journal of the American Association for Cancer Research.

[19] Pound CR, Partin AW, Epstein JI, Walsh PC (1997). Prostate-specific antigen after anatomic radical retropubic prostatectomy. Patterns of recurrence and cancer control. The Urologic Clinics of North America.

[20] Thompson IM, Tangen CM, Paradelo J, Lucia MS, Miller G, Troyer D, Messing E, Forman J, Chin J, Swanson G, Canby-Hagino E, Crawford ED (2009). Adjuvant radiotherapy for pathological T3N0M0 prostate cancer significantly reduces risk of metastases and improves survival: long-term follow-up of a randomized clinical trial. The Journal of Urology.

[21] Cullen J, Rosner IL, Brand TC, Zhang N, Tsiatis AC, Moncur J, Ali A, Chen Y, Knezevic D, Maddala T, Lawrence HJ, Febbo PG, Srivastava S (2015). A biopsy-based 17-gene genomic prostate score predicts recurrence after radical prostatectomy and adverse surgical pathology in a racially diverse population of men with clinically low- and intermediate-risk prostate cancer. European Urology.

[22] Klein EA, Cooperberg MR, Magi-Galluzzi C, Simko JP, Falzarano SM, Maddala T, Chan JM, Li J, Cowan JE, Tsiatis AC, Cherbavaz DB, Pelham RJ, Tenggara-Hunter I (2014). A 17- gene assay to predict prostate cancer aggressiveness in the context of Gleason grade heterogeneity, tumor multifocality, and biopsy undersampling. European Urology.

[23] Cuzick J, Swanson GP, Fisher G, Brothman AR, Berney DM, Reid JE, Mesher D, Speights VO, Stankiewicz E, Foster CS, Moller H, Scardino P, Warren JD, Transatlantic Prostate G (2011). Prognostic value of an RNA expression signature derived from cell cycle proliferation genes in patients with prostate cancer: a retrospective study. The Lancet Oncology.

[24] Ross AE, Feng FY, Ghadessi M, Erho N, Crisan A, Buerki C, Sundi D, Mitra AP, Vergara IA, Thompson DJ, Triche TJ, Davicioni E, Bergstralh EJ (2014). A genomic classifier predicting metastatic disease progression in men with biochemical recurrence after prostatectomy. Prostate Cancer Prostatic Dis.

[25] Cooperberg MR, Davicioni E, Crisan A, Jenkins RB, Ghadessi M, Karnes RJ (2015). Combined value of validated clinical and genomic risk stratification tools for predicting prostate cancer mortality in a high-risk prostatectomy cohort. European Urology.

[26] Erho N, Crisan A, Vergara IA, Mitra AP, Ghadessi M, Buerki C, Bergstralh EJ, Kollmeyer T, Fink S, Haddad Z, Zimmermann B, Sierocinski T, Ballman KV (2013). Discovery and validation of a prostate cancer genomic classifier that predicts early metastasis following radical prostatectomy. PLoS One.

[27] Karnes RJ, Bergstralh EJ, Davicioni E, Ghadessi M, Buerki C, Mitra AP, Crisan A, Erho N, Vergara IA, Lam LL, Carlson R, Thompson DJ, Haddad Z (2013). Validation of a genomic classifier that predicts metastasis following radical prostatectomy in an at risk patient population. J Urol.

[28] Den RB, Feng FY, Showalter TN, Mishra MV, Trabulsi EJ, Lallas CD, Gomella LG, Kelly WK, Birbe RC, McCue PA, Ghadessi M, Yousefi K, Davicioni E (2014). Genomic prostate cancer classifier predicts biochemical failure and metastases in patients after postoperative radiation therapy. Int J Radiat Oncol Biol Phys.

[29] Cooperberg MR, Hilton JF, Carroll PR (2011). The CAPRA-S score: A straightforward tool for improved prediction of outcomes after radical prostatectomy. Cancer.

[30] Greene KL, Meng MV, Elkin EP, Cooperberg MR, Pasta DJ, Kattan MW, Wallace K, Carroll PR (2004). Validation of the Kattan preoperative nomogram for prostate cancer recurrence using a community based cohort: results from cancer of the prostate strategic urological research endeavor (capsure). The Journal of Urology.

[31] Brockman JA, Alanee S, Vickers AJ, Scardino PT, Wood DP, Kibel AS, Lin DW, Bianco FJ, Rabah DM, Klein EA, Ciezki JP, Gao T, Kattan MW (2015). Nomogram predicting prostate cancer-specific mortality for men with biochemical recurrence After radical prostatectomy. European Urology.

[32] Nielsen T, Wallden B, Schaper C, Ferree S, Liu S, Gao D, Barry G, Dowidar N, Maysuria M, Storhoff J (2014). Analytical validation of the PAM50-based Prosigna Breast Cancer Prognostic Gene Signature Assay and nCounter Analysis System using formalin-fixed paraffin-embedded breast tumor specimens. BMC Cancer.

[33] Kaklamani V (2006). A genetic signature can predict prognosis and response to therapy in breast cancer: Oncotype DX. Expert Rev Mol Diagn.

[34] Foster JM, Oumie A, Togneri FS, Vasques FR, Hau D, Taylor M, Tinkler-Hundal E, Southward K, Medlow P, McGreeghan-Crosby K, Halfpenny I, McMullan DJ, Quirke P, Keating KE, Griffiths M, Spink KG, Brew F (2015). Cross-laboratory validation of the OncoScan(R) FFPE Assay, a multiplex tool for whole genome tumour profiling. BMC Medical Genomics.

[35] Chitale D, Gong Y, Taylor BS, Broderick S, Brennan C, Somwar R, Golas B, Wang L, Motoi N, Szoke J, Reinersman JM, Major J, Sander C (2009). An integrated genomic analysis of lung cancer reveals loss of DUSP4 in EGFR-mutant tumors. Oncogene.

[36] Gao J, Aksoy BA, Dogrusoz U, Dresdner G, Gross B, Sumer SO, Sun Y, Jacobsen A, Sinha R, Larsson E, Cerami E, Sander C, Schultz N (2013). Integrative analysis of complex cancer genomics and clinical profiles using the cBioPortal. Sci Signal.

[37] Cerami E, Gao J, Dogrusoz U, Gross BE, Sumer SO, Aksoy BA, Jacobsen A, Byrne CJ, Heuer ML, Larsson E, Antipin Y, Reva B, Goldberg AP, Sander C, Schultz N (2012). The cBio cancer genomics portal: an open platform for exploring multidimensional cancer genomics data. Cancer Discov.

[38] Milioli HH, Vimieiro R, Riveros C, Tishchenko I, Berretta R, Moscato P (2015). The discovery of novel biomarkers improves breast cancer intrinsic subtype prediction and reconciles the labels in the METABRIC data set. PLoS One.

[39] Pereira B, Chin SF, Rueda OM, Vollan HK, Provenzano E, Bardwell HA, Pugh M, Jones L, Russell R, Sammut SJ, Tsui DW, Liu B, Dawson SJ (2016). The somatic mutation profiles of 2,433 breast cancers refines their genomic and transcriptomic landscapes. Nat Commun.

