# Supplementary material for: Robust genomic copy number predictor of pan cancer metastasis
Source: Genes Cancer. 2018 Jan;9(1-2):66–77. doi: 10.18632/genesandcancer.165 (PMC5931251; doi:10.18632/genesandcancer.165)
Supplement: Supplementary file 1 [file ganc-09-066-s001.pdf]

Robust Genomic Copy Number Predictor of Pan Cancer Metastasis – Pearlman et al

S1 Table. Left and Center: Logistic regression and Cox proportional hazards models predict progression to metastasis for full set (variable number by cohort) and panMPS (295) genes. Right: Linear regression model predicting correlation (r^2) between MPS and panMPS.

Cohort	Variables	Logistic Regression				Cox Regression				Linear Regression
		Odds Ratio	P	95% CI	AUC	Hazard Ratio	95% CI	Conc-index	P	r^2
MSK_Prostate	MPS (320 genes)	5.39	0.001	2.03 to15.49	0.69	4.85	2.00 to 11.77	0.72	0.0005	0.97
	panMPS	6.01	0.001	2.21 to 17.89	0.71	5.42	2.18 to 13.49	0.74	0.0003	0.97
Duke_Prostate	MPS (351 genes)	10.99	0.004	2.36 to 65.88	0.72	3.54	1.20 to 10.44	0.62	0.02	0.99
	panMPS	11.39	0.004	2.39 to 70.36	0.72	3.41	1.15 to 10.12	0.62	0.03	0.97
Montefiore_TNBC	MPS (352 genes)	44.36	0.02	2.87 to 2005.4	0.75	4.01	1.04 to 15.44	0.59	0.04	0.99
	panMPS	44.74	0.02	2.91 to 1927.9	0.75	4.06	1.03 to 16.04	0.59	0.05	0.96
MSK_Lung	MPS (353 genes)	3.78×10^3	0.006	42.79 to 1.04×10^7	0.94	4.65	1.04 to 20.63	0.65	0.04	0.96
	panMPS	3.45×10^3	0.006	41.5 to1.26×10^7	0.94	6.57	1.31 to 33.04	0.67	0.02	0.96

S2 Table. Logistic regression models predicting progression to metastasis for prostate cancer based on panMPS and clinical variables

Cohort	MSK Prostate CA (n=182, mPT=25, iPT=157)				Duke Prostate CA (n=61, mPT=37, iPT=24)			
**Variable**	**Odds Ratio**	**P**	**95% CI**	**AUC**	**Odds Ratio**	**P**	**95% CI**	**AUC**
**Univariate**								
panMPS	5.98	0.001	2.12 to 18.57	0.7	11.84	0.002	2.78 to 67.71	0.75
Preop PSA	1.06	0.02	1.02 to 1.11	0.66	1.1	0.08	1.01 to 1.21	0.61
Biopsy Gleason	3.82	0.02	1.21 to 11.10	0.58	3.96	0.09	0.89 to 27.84	0.59
Clinical Stage	2.3	0.06	0.98 to 5.59	0.6	4	0.1	0.86 to 28.88	0.61
Path Gleason	68	1.3×10^{-10}	20.51 to 280.1	0.81	7.5	0.01	1.83 to 51.3	0.66
Path Stage	5.19	0.001	2.12 to 14.08	0.7	0.47	0.23	0.13 to 1.63	0.57
%Genome Inst.	1.17	1.4×10^{-5}	1.09 to 1.26	0.74	1.04	0.12	1.00 to 1.12	0.8
**Multivariate**								
panMPS	4.09	0.01	1.38 to 13.19	0.75	14.32	0.003	2.7 to 103.6	0.78
Preop PSA	1.04	0.05	1.01 to 1.1		1.1	0.06	1.01 to 1.24	
panMPS	5.09	0.003	1.8 to 15.54	0.73	6.06	0.06	2.4 to 70.4	0.68
Biopsy Gleason	2.32	0.15	0.69 to 7.17		1.18	0.88	1.03 to 47.04	
panMPS	5.51	0.001	1.99 to 16.59	0.73	4.42	0.13	0.71 to 34.06	0.72
Clinical Stage	1.93	0.15	0.79 to 4.83		2.76	0.26	0.52 to 21.2	
panMPS	1.5	0.55	0.39 to 5.88	0.86	7.45	0.03	1.4 to 49.38	0.77
Path Gleason	56.8	1.21×10^{-8}	15.57 to 263.14		4.83	0.06	1.1 to 34.44	
panMPS	3.93	0.01	1.43 to 11.94	0.77	10.59	0.01	2.18 to 65.6	0.73
Path Stage	3.83	0.006	1.49 to 10.74		0.59	0.45	0.15 to 2.32	
panMPS	1.79	0.32	0.59 to 5.97	0.75	8,45	0.03	1.41 to 62.2	0.73
%Genome Inst.	1.15	0.001	1.06 to 1.25		1.01	0.53	0.98 to 1.07	
panMPS	1.49	0.52	0.45 to 5.39	0.8	9.93	0.02	1.47 to 85.45	0.78
Preop PSA	1.03	0.21	1.00 to 1.09		1.1	0.06	1.01 to 1.24	
%Genome Inst.	1.14	0.004	1.05 to 1.25		1.02	0.5	0.98 to 1.08	
panMPS	3.7	0.03	1.2 to 11.93	0.76	8.6	0.04	1.25 to 90.53	0.74
Biopsy Gleason	2.2	0.21	0.59 to 7.1		1.35	0.78	0.17 to 13.8	
Preop PSA	1.04	0.05	1.01 to 1.09		1.11	0.09	1.01 to 1.28	
panMPS	4.71	0.01	1.66 to 14.48	0.75	4.47	0.13	0.70 to 36.93	0.71
Biopsy Gleason	2.24	0.17	0.66 to 6.97		0.93	0.95	0.12 to 8.55	
Clinical Stage	1.88	0.17	0.77 to 4.76		2.8	0.26	0.51 to 22.16	
panMPS	1.29	0.71	0.34 to 4.99	0.85	6.88	0.03	1.27 to 45.95	0.77

Path Gleason	47.21	8.69×10^{-8}	12.71 to 224.2		4.91	0.06	1.08 to 35.13	
Path Stage	2.87	0.08	0.87 to 9.88		0.56	0.42	1.13 to 2.31	
panMPS	3.56	0.03	1.16 to 11.62	0.78	6.97	0.04	0.94 to 77.9	0.76
Biopsy Gleason	2.15	0.22	0.59 to 6.97		1.07	0.08	0.11 to 11.94	
Clinical Stage	1.41	0.48	0.54 to 3.71		2.73	0.46	0.46 to 22.54	
Preop PSA	1.04	0.07	1.01 to 1.08		1.11	0.29	1.01 to 1.29	
panMPS	3.5	0.03	1.16 to 11.2	0.79	5.68	0.13	0.66 to 68.5	0.76
Biopsy Gleason	2.2	0.22	0.60 to 7.51		1.34	0.82	0.12 to 31.9	
Clinical Stage	1.37	0.52	0.52 to 3.61		2.39	0.37	0.38 to 20.3	
Preop PSA	1.04	0.06	1.01 to 1.09		1.1	0.13	0.99 to 1.27	
Age	0.51	0.35	0.13 to 2.49		7.85×10^{6}	0.99	3.85×10^{-104} to NA	

S3 Table. Cox proportional hazards model of panMPS and its association with metastasis-free survival for prostate cancer based on panMPS and clinical variables

Cohort	MSK Prostate CA (n=222, mPT=25, iPT=197)				Duke Prostate CA (n=76, mPT=37, iPT=39)			
Variables	Hazard Ratio	95% CI	Conc -indx	P	Hazard Ratio	95% CI	Conc -indx	P
**Univariate**								
panMPS	4.2	1.67 to 10.4	0.7	0.002	3.9	1.48 to 10.4	0.63	0.01
Preop PSA	1.01	1.00 to 1.01	0.63	7.60×10^{-5}	1	0.97 to 1.03	0.51	0.98
Biopsy Gleason	3.11	1.24 to 7.83	0.73	0.02	1.63	0.71 to 3.72	0.65	0.25
Clinical Stage	1.75	0.78 to 3.91	0.65	0.17	0.48	0.21 to 1.09	0.64	0.08
Path Gleason	17.34	7.63 to 39.4	0.97	9.90×10^{-12}	1.67	0.84 to 3.34	0.71	0.14
Path Stage	3.99	1.67 to 9.58	0.82	0.002	0.61	0.23 to 1.57	0.57	0.3
%Genome Inst.	1.11	1.07 to 1.16	0.67	3.30×10^{-7}	1.01	0. 99 to 1.02	0.66	0.2
**Multivariate**								
panMPS	4.31	1.72 to 10.8	0.74	0.002	3.52	1.18 to 10.5	0.71	0.02
Preop PSA	1.01	1.00 to 1.01		0.0002	0.99	1.02 to 1.12		0.7
panMPS	3.77	1.49 to 9.51	0.72	0.005	3.13	1.06 to 9.29	0.66	0.04

Biopsy Gleason	2.18	0.83 to 5.69		0.1	1.86	0.88 to 3.93		0.1
panMPS	4.59	1.83 to 11.6	0.71	0.001	2.25	0.62 to 8.17	0.6	0.22
Clinical Stage	1.81	0.81 to 4.03		0.15	1.86	0.79 to 4.34		0.15
panMPS	1.79	0.69 to 4.65	0.86	0.23	2.97	0.94 to 9.41	0.64	0.06
Path Gleason	14.34	5.96 to 34.5		2.74×10^{-9}	1.29	0.62 to 2.69		0.5
panMPS	2.96	1.19 to 7.33	0.76	0.02	3.19	1.05 to 9.64	0.62	0.04
Path Stage	3.03	1.22 to 7.55		0.02	0.73	0.27 to 1.91		0.5
panMPS	1.37	0.47 to 3.97	0.71	0.56	3.26	1.08 to 9.8	0.62	0.04
%Genome Inst.	1.1	1.04 to 1.16		0.001	1.01	0.99 to 1.02		0.32
panMPS	1.29	0.44 to 3.85	0.73	0.64	3.35	1.1 to 10.17	0.62	0.03
Preop PSA	1.01	1.00 to 1.01		0.0001	0.99	0.97 to 1.02		0.77
%Genome Inst.	1.09	1.04 to 1.16		0.001	1.01	0.99 to 1.02		0.35
panMPS	3.69	1.44 to 9.47	0.76	0.01	2.75	0.78 to 9.64	0.67	0.11
Biopsy Gleason	2.3	0.86 to 5.94		0.09	1.19	0.49 to 2.92		0.69
Prep PSA	1.01	1.00 to 1.01		0.0001	1.06	1.01 to 1.11		0.03
panMPS	4.07	1.58 to 10.5	0.74	0.01	1.96	0.53 to 7.23	0.6	0.31
Biopsy Gleason	2.09	0.81 to 5.42		0.13	1.69	0.72 to 3.97		0.23
Clinical Stage	1.76	0.78 to 3.94		0.17	2.02	0.84 to 4.85		0.11
panMPS	1.79	0.71 to 4.51	0.86	0.22	2.67	0. 56 to 7.6	0.64	0.1
Path Gleason	11.98	4.98 to 28.8		2.91×10^{-8}	1.35	0.49 to 3.14		0.44
Path Stage	2.04	0.83 to 5.06		0.12	1.91	0.78 to 4.64		0.46
panMPS	3.95	1.51 to 10.3	0.76	0.01	2.06	0.56 to 7.61	0.65	0.28
Biopsy Gleason	2.19	0.84 to 5.72		0.11	1.25	0.49 to 3.14		0.63
Clinical Stage	1.57	0.68 to 3.58		0.29	1.91	0.78 to 4.64		0.15
Preop PSA	1.01	1.00 to 1.01		0.001	1.05	1.01 to 1.11		0.04
panMPS	3.95	1.49 to 10.5	0.76	0.01	1.73	0.46 to 6.55	0.68	0.42
Biopsy Gleason	2.21	0.84 to 5.76		0.11	1.58	0.59 to 3.92		0.37
Clinical Stage	1.56	0.68 to 3.6		0.29	1.68	0.66 to 4.29		0.27
Preop PSA	1.01	1.00 to 1.01		0.001	1.04	0.99 to 1.11		0.13

Age	0.89	0.26 to 3.09	0.86	8.31×10^7	0 to Inf	0.99

S4 Table. Clump analysis of genes, including Z_{genes} score, clump index, number of genes in clump and PubMed ID for metastasis function annotations, metastasis predictive biomarker and metastasis in the title.

Gene	Z_{genes} score	clump index	No. of genes in clump	Metastasis Function PubMed ID	Metastasis Predictive Biomarkers PubMed ID	Metastasis PubMed ID
ACTL8	1.9	1	2			23592437
ARHGEF10L	2.1	1	2			
LEPREL1	2.6	2	2	24319452		
TP63	3	2	2	21760596, 24488880, 25376609*	15761962, 23913939	19142959, 26208975
GLRB	2.7	3	2			
GRIA2	2.3	3	2	18953328*	18953328	
CCDC125	2	4	7	28374495*		
CDK7	2.7	4	7	23393140, 25490451, 25820824		25117707
CENPH	1.9	4	7		22999412	
MARVELD2	3	4	7			
MRPS36	2.6	4	7			
RAD17	2.6	4	7			
TAF9	2.6	4	7			
EPHA7	1.8	5	2			16007213
MAP3K7	3.2	5	2	23370768, 25770290, 27066479*		17785553
ASCC3	1.8	6	2			
SIM1	2.2	6	2			
EPM2A	2.4	7	2			18824542
UTRN	2.3	7	2			
C6orf118	2.8	8	2			
PDE10A	4.7	8	2			
CLIP2	3.1	9	4			
EIF4H	2	9	4			
LAT2	2	9	4	24505132*		
RFC2	1.8	9	4			
MDH2	2	10	3			
STYXL1	2.3	10	3			
TMEM120A	1.7	10	3			
PILRA	1.9	11	2			

PILRB	2.9	11	2	20065029*		
ACTL6B	1.7	12	9			21136598
AGFG2	2.3	12	9			
C7orf51	2.2	12	9			
FBXO24	2.5	12	9			
LRCH4	2.2	12	9			
MOSPD3	2.3	12	9			
PCOLCE	1.8	12	9			
TFR2	2.6	12	9			
TSC22D4	2.1	12	9			
COPG2	3.1	13	7			
CPA1	2.1	13	7			
CPA2	2.1	13	7			
CPA4	1.9	13	7			27073726
CPA5	2.8	13	7			
MEST	3.2	13	7	27987347*	23229728	
TSGA14	9.4	13	7			
CSMD1	4.6	14	2	18614856		
MYOM2	2.1	14	2			
ERI1	1.9	15	2			
MFHAS1	3.3	15	2			
MSRA	5.1	16	2	15638783*		17784942
TNKS	4	16	2			
C8orf16	2.2	17	2			
MTMR9	1.9	17	2			
GATA4	3.2	18	2	19509152, 26473289*	20222162	23239811, 24862985
NEIL2	2.7	18	2	25267977*		
C8orf79	2.9	19	2			
DLC1	6.5	19	2	11118037		
SGCZ	8.2	20	2			
TUSC3	3.1	20	2	23404293, 24096664, 24435307, 22312193*	23096450	
MTMR7	3.7	21	4			
MTUS1	3.6	21	4	19794912, 24299308, 25885343, 25586720*		16650523
PDGFRL	4.8	21	4	17610272*		
SLC7A2	4.1	21	4	27783672*		
ASAH1	7.1	22	2	23423838		
PCM1	1.7	22	2			

NAT2	3.3	23	3			
PSD3	7.3	23	3			
SH2D4A	2.9	23	3			
FAM160B2	4.3	24	5			
HR	2.7	24	5			
LGI3	2	24	5			
NUDT18	2.3	24	5			
REEP4	2.5	24	5			
BMP1	2.2	25	3		19723875	23584484
PHYHIP	2.2	25	3			
POLR3D	2.7	25	3			
BIN3	4.5	26	9			
C8orf58	3	26	9			
EGR3	2	26	9	22949304*		23342084
KIAA1967	5.9	26	9			
PDLIM2	2.9	26	9	23584482		24196835
PPP3CC	5.7	26	9			
RHOBTB2	1.7	26	9	20930524, 21801820, 18762809*	15922864, 19173804, 19937980	
SLC39A14	4	26	9			
SORBS3	3	26	9			
CHMP7	2.4	27	3			
TNFRSF10A	2.2	27	3			
TNFRSF10D	1.9	27	3	22509103*		
ENTPD4	2.7	28	2			
LOXL2	1.9	28	2	25128648, 24014025, 24008674, 23971878, 27063404*	27008697	23030485
CDCA2	2	29	3	23418564		17611626
EBF2	5.1	29	3			19671856
KCTD9	2	29	3			
ADRA1A	3.9	30	7		21360566	24607627, 26276037
CHRNA2	3.5	30	7			
DPYSL2	3.3	30	7			
EPHX2	3.3	30	7	21059750*		16456776
PTK2B	7	30	7			
STMN4	3.3	30	7			
TRIM35	2.6	30	7			
C8orf80	3.6	31	3			

ELP3	3.4	31	3		22740850	
SCARA5	3.3	31	3	20038795, 24061576	22642751	
HMBOX1	1.8	32	2			
KIF13B	2.7	32	2			
C8orf34	6	33	7			
CPA6	3.8	33	7			
NCOA2	5.6	33	7			25295534
PRDM14	4.7	33	7	21339739, 25233927, 25635434	17942894, 23690269	
PREX2	7.5	33	7		22622578, 25151370, 25829446	
SLCO5A1	9.1	33	7			
SULF1	8.6	33	7		19780053, 21228115, 22653794	19373441
EYA1	3.4	34	6	28087632*		24729159
KCNB2	6.8	34	6			
LACTB2	2.6	34	6			
MSC	2	34	6			
TRPA1	2.3	34	6	24037916, 28126501*		
XKR9	2.7	34	6			
CRISPLD1	4.9	35	6			
GDAP1	2	35	6			
HNF4G	4.1	35	6			
JPH1	6	35	6			
PI15	3	35	6			
ZFHX4	4.3	35	6			
HEY1	3	36	2	22864395*	23226563	
STMN2	2.8	36	2			
PAG1	2	37	2	21092590, 21156787, 25692234*		20388373, 21388951, 24675741
ZNF704	2.5	37	2			
CNBD1	7.6	38	2			
CNGB3	1.8	38	2			
PTDSS1	2.5	39	2			
SDC2	3.4	39	2	20863401, 22745764,19292938*	19288017, 20683009	
GRHL2	2.4	40	4	18752864, 20938050, 23284647, 23814079, 24756066	23441166, 26355710	

NCALD	8.4	40	4			27027352
YWHAZ	2.2	40	4	20098429, 22912335, 25895496*		
ZNF706	2.8	40	4			
DPYS	3.2	41	3			
LRP12	2	41	3		22138261, 14676824	
ZFPM2	10.5	41	3			
ANGPT1	2.4	42	2	28380526*		20651738
RSPO2	1.8	42	2	21732367, 25769727	26416247	24476626
CSMD3	9.1	43	2			
TRPS1	5.6	43	2	24709795, 26183398	16043716, 23762846, 26377811	
MYC	4.2	44	3	20133671, 27210806*		15810077, 9012485
POU5F1B	2.9	44	3			
TMEM75	3.5	44	3			
ADCY8	8.5	45	5	20505109*		19082487, 22419659
ASAP1	3.6	45	5	18519696, 20154719, 23811350*	24427349, 24788532	
EFR3A	3.1	45	5			
KCNQ3	6	45	5			
OC90	1.9	45	5			
PHF20L1	2.8	46	5			
SLA	2.2	46	5	28261115*		
TG	3.8	46	5			
TMEM71	2.9	46	5			
WISP1	2.2	46	5	19078974, 21109017, 21453685, 24239602*	17579708, 20372786	
CDC42BPG	2.3	47	2			
MEN1	2.1	47	2	28106886*		
ESD	2.6	48	3	21596165		
HTR2A	3.3	48	3	16873768*		
LRCH1	2.4	48	3	28028151*		
DACH1	1.9	49	3	16980615, 18467491*		
KLHL1	1.9	49	3			
PCDH9	4.5	49	3	25172662	22300792, 25869928, 25979483	
DDX19A	2.3	50	2			
ST3GAL2	2.4	50	2			

BCAR1	2.5	51	2	22476538, 24962474*	10639513, 15448007, 17192874, 23904007	15972849, 21765937, 22241677
CFDP1	3	51	2			
ADAMTS18	3.5	52	4	18449890	25569086	21196270, 21047771, 24896365
CLEC3A	2.3	52	4			19173304
NUDT7	2.2	52	4			
WWOX	9.3	52	4	14695174, 18487609, 23824713	15073846, 16360296, 17289881, 21731849	
BCMO1	6.5	53	7	23803888		
C16orf46	2.2	53	7			
GAN	5.1	53	7	25757924*		
GCSH	1.9	53	7			
HSD17B2	2.6	53	7		25929810	
PKD1L2	6.9	53	7			
PLCG2	5.6	53	7	24092775*		
HSDL1	2	54	3			
LRRC50	2.4	54	3			
MBTPS1	2.7	54	3			
ATP2C2	3.6	55	3			
KIAA1609	1.9	55	3			
WFDC1	2.3	55	3	18842679, 19488830, 25219356*	10967136, 12032731, 15305341	
CRISPLD2	5.4	56	5			
KIAA0513	2.1	56	5			
KLHL36	1.8	56	5			
USP10	2.3	56	5	24332849, 25168367	16773218	24343337
ZDHHC7	2.5	56	5			
C16orf74	2.3	57	8		21203532	
COX4I1	2.6	57	8			
COX4NB	2.5	57	8			
FOXF1	2.2	57	8	24186199, 21341990*	23103611	20145151, 20587515, 23864317
GINS2	2.1	57	8	21082043, 24273450	24137407, 25348432	
IRF8	2.5	57	8	23308054, 25495942*	24091328	17409439, 19074829, 24175153
KIAA0182	2	57	8			

MTHFSD	2.4	57	8			
BANP	5.9	58	13	17668048, 25086032		20709157, 18981184, 18822384
C16orf85	2.1	58	13			
CA5A	6.4	58	13			
CYBA	2.9	58	13			
IL17C	1.8	58	13	27365405*		
JPH3	4.8	58	13			
KLHDC4	2.5	58	13	27030985		
MVD	2.3	58	13			
RNF166	2.2	58	13			
SLC7A5	4.7	58	13	21439283, 17609412*	23981989, 26244545	
SNAI3	2.4	58	13			
ZC3H18	2.1	58	13			
ZFPM1	2.5	58	13			
CDT1	2	59	2		26408331	21159650
FAM38A	2.7	59	2	22792288		
CBFA2T3	1.9	60	3	12183414		25749032
GALNS	2.3	60	3			
TRAPPC2L	1.9	60	3			
ANKRD11	6.7	61	2	18840648		21986947
CDH15	1.9	61	2			9615235
FANCA	1.9	62	4	25015289*		
SPIRE2	1.7	62	4			
TCF25	2.1	62	4			
TUBB3	2.6	62	4	25414139	24926347	20534991, 24053422
AFG3L1	2.3	63	2			
DEF8	1.9	63	2			
DHX58	8.9	64	5			
HSPB9	2.9	64	5			
KAT2A	3.2	64	5	26278503*		
KCNH4	3.8	64	5			
RAB5C	3.5	64	5			
ASPSCR1	1.8	65	2			
NOTUM	2.7	65	2			
DUS1L	2.3	66	2			
FASN	3	66	2	18770866, 22266115, 22892389, 27769711*		17882277
DTNA	2.2	67	2			

NOL4	3.9	67	2			
C19orf57	2.8	68	2			
CC2D1A	4	68	2			
AR	4.9	69	4	26701731*	7541709, 8604394	7723794, 11325816,
EDA2R	2	69	4			
HEPH	1.8	69	4			
OPHN1	5.8	69	4			
ALCAM	2.5	NA	1	16204050, 15140234, 24091017*	15509676, 10702391	9502422, 15986133, 16024937, 11206837, 18202807
ANXA13	2.1	NA	1		22294041	22559327
ARHGEF10	2.9	NA	1			21412932
ARHGEF5	2.7	NA	1			21525037
ATP6V1C1	2.4	NA	1	24155661, 24454753	20404513	15558013, 19885577, 18638373, 19424568, 17467328
BFSP2	2.4	NA	1			
BLK	2.1	NA	1	27601209*		
BOD1L	2.4	NA	1			
C13orf23	2.2	NA	1			
C16orf80	2.2	NA	1			
CCDC25	4.4	NA	1		22202459	
CD226	3.3	NA	1	24468679, 25209846*		20008292
CDH13	10.9	NA	1	25965835*		11389090, 12067979, 16807071, 15245595, 20642860
CDH17	2.8	NA	1	19676131, 20568120, 23298905, 23604127, 23554857, 23479506*	23326130, 22904132	
CDH2	3.4	NA	1	19190132, 21906588*		20848731
CDH8	3.7	NA	1			
CDYL2	2.5	NA	1			
CLCNKB	2	NA	1			
CLDN3	2.6	NA	1	19208807		
CNGB1	1.8	NA	1			
CNTNAP4	3.2	NA	1			
COL11A1	1.9	NA	1			11375892, 19112599, 21047417
COL12A1	1.8	NA	1	21462330		
COL19A1	3.4	NA	1			

COL21A1	1.8	NA	1			
CTNNA2	1.8	NA	1	23700281*		24100690
CTSB	2.8	NA	1	16707449, 20133781, 23986436*		
CYP7B1	1.7	NA	1		17639508	
DCC	6.6	NA	1	26345965		9387268
DCHS2	2.8	NA	1			24898286
DGKG	1.9	NA	1			
DIAPH3	3.3	NA	1	22593025, 28115158		
DLGAP2	2.2	NA	1			
DNAH2	1.8	NA	1			
DOCK5	5.4	NA	1			
DPYD	2.9	NA	1			
ENOX1	5.6	NA	1			21055930
EPO	2	NA	1	22019117*		24497137
FBXL18	1.9	NA	1			
FBXL4	1.7	NA	1			
FSTL5	2.2	NA	1			
GABRA2	2.3	NA	1			
GAS8	2.9	NA	1			
GHDC	1.8	NA	1			
GIGYF1	2.7	NA	1			
GLG1	2.1	NA	1	25301730	19148508	
GPC5	2.1	NA	1	23962560	26631038	24260047,25093697, 25818666, 26098560
GRID2	5.1	NA	1			
GRK5	2.4	NA	1	22099983, 24755472, 24632620*		
GRM1	1.9	NA	1	18435704, 23085756, 24491800, 16040064		
GYS2	2.8	NA	1			
HIP1	4.4	NA	1	12163454	21697888	26595459
IMPA1	1.9	NA	1			
IQCE	1.8	NA	1			
KALRN	2.4	NA	1			
KCNAB1	5.8	NA	1			
KCTD8	2.8	NA	1			
KIAA0196	2.6	NA	1		16130124	
LPHN3	2.5	NA	1		23317273	

LZTS1	2	NA	1	18559591	18686028, 24466374	11410489,23695671, 24525428
MACROD1	4.8	NA	1			
MDGA2	2.8	NA	1			
ME1	2.5	NA	1	25753478		
MECOM	2	NA	1			
MEF2C	2.3	NA	1	19584403, 26364852*		
MEIS2	3.9	NA	1	21617755*		
MEPCE	2.1	NA	1			
MLYCD	2.4	NA	1			
MMP16	3.5	NA	1			21600596
MTDH	1.9	NA	1	19111877, 21976539, 21371176, 24099913		19723648, 22031094, 23851509
MTMR9	1.9	NA	1			
MYLK	2.8	NA	1		15970650, 25179839	
NALCN	2.2	NA	1			
NECAB2	2	NA	1			
NFAT5	2.3	NA	1	27875309*		19011242, 22266867, 25152734, 25311085, 26299924
NIPAL2	1.9	NA	1			
NKIRAS2	2	NA	1			
NKX2-6	2.4	NA	1			
NLGN4Y	2.4	NA	1			
NRXN1	3.2	NA	1			
NUS1	2.2	NA	1			
PDS5B	2	NA	1		23850494	
PKIA	3.3	NA	1			
PLCB1	1.9	NA	1	25823422*	26620550	
PPM1L	2	NA	1			
PPP2R5B	1.8	NA	1			
PTK2	2.3	NA	1	14578863, 27789711*		
RAB9A	3.7	NA	1			
RALYL	2.8	NA	1			
RCOR2	1.7	NA	1			
RFX1	2.2	NA	1			
RGS22	2.7	NA	1	21533872		26323264
RIMS2	6	NA	1			
RNF40	2.1	NA	1		22155569	

RPL7	2.3	NA	1			
SF1	2.5	NA	1	11119501*	18824868	
SLC26A7	2.2	NA	1			
SLC9A9	2.7	NA	1		25835977	
SMARCB1	1.8	NA	1		15899790, 16528370, 17040295, 21057957, 24503755	
STAG3	2.4	NA	1			
STAU2	4.6	NA	1			
STIP1	1.8	NA	1		24163084, 24488757	
STK3	1.8	NA	1			
STX1A	2.2	NA	1	25530917*		
TBC1D10B	1.8	NA	1			
TBC1D22A	4.6	NA	1	24080422*		
TCEB1	1.8	NA	1	18844214		25676555
TFDP1	2.3	NA	1	14618416	26684807	19995430
TFE3	2.1	NA	1	23226201*	11438465, 12459622, 19606011, 20154303, 20871214	
TICAM2	1.8	NA	1	25941940*		
TOX	4.3	NA	1			
TRDN	3	NA	1			
UBE2CBP	2.8	NA	1			
UBR5	2.2	NA	1			
VPS13B	3.9	NA	1			
VPS13C	1.8	NA	1			
WDR59	2.1	NA	1			
WDR7	2	NA	1			
WWP2	1.8	NA	1	26662306*		23938591, 26783238
XPO7	2.3	NA	1			
YWHAG	2.7	NA	1			
ZBTB20	1.8	NA	1	25311537	21702992	
ZFAT	2.5	NA	1			
ZFHX3	2.2	NA	1			
ZHX2	2.6	NA	1		17447851	
ZSWIM4	2.8	NA	1			

S5A Table. Genes in gene set for Metastasis ID
SEPT9,DEC1,ABAT,ABCA13,ABCA2,ABCA3,ABCB1,ABCB11,ABCB5,ABCC10,ABCC2, ABCC3,ABCC5,ABCD1,ABCD3,ABCE1,ABCG1,ABCG2,ABCG4,ABHD11,ABHD4,ABI1,ABI2, ABL1,ABL2,ABR,ACACA,ACCS,ACE,ACE2,ACHE,ACP1,ACP5,ACP6,ACPT,ACR,ACRBP, ACSS2,ACTB,ACTG2,ACTN4,ADAM10,ADAM19,ADAM29,ADAM33,ADAM7,ADAM8,ADAM9,ADAMTS1,ADAMTS12,ADAMTS13,ADAMTS18,ADAMTS19,ADAMTS5,ADAMTS8,ADIPOQ, ADIPOR1,ADIPOR2,ADM,ADO,ADORA2B,ADRA2A,ADRB2,ADRBK2,ADRM1,AES,AFAP1, AFAP1L1,AFM,AFP,AGA,AGBL2,AGK,AGR2,AGR3,AGXT,AHNAK,AHR,AHSG,AIM1,AIM2, AKAP12,AKAP13,AKIRIN2,AKR1B10,AKR1C1,AKR1C3,AKT1,AKT2,AKT3,ALDH1A1, ALDH1A3,ALDH3A1,ALK,ALCAM,ALKBH5,ALOX5,AMN,AMOTL1,AMOTL2,ANG,ANGPT2, ANGPTL2,ANGPTL3,ANLN,ANKRD11,ANO1,ANO9,ANP32A,ANTXR1,ANXA1,ANXA11, ANXA2,ANXA5,ANXA7,APAF1,APC,APCS,API5,APLP2,APOA1,APOBEC3G,APOH,APP, APPL1,AQP1,AQP3,AQP5,AQP6,AQP9,AR,ARAF,AREG,ARF1,ARF6,ARFGAP1,ARG1, ARHGAP21,ARHGAP5,ARHGAP6,ARHGEF3,ARHGEF5,ARHGEF7,ARID1A,ARID2,ARID3B, ARID4A,ARID4B,ARL4C,ARMC3,ARNT,ARPC1B,ARRB1,ARRDC3,ASAP1,ASCL2,ASIP,ASL,ASPM,ASS1,ATAD2,ATF1,ATF3,ATF4,ATF5,ATF6,ATG10,ATG16L1,ATG4A,ATG5,ATIC, ATM,ATOH1,ATOH8,ATP11A,ATP4A,ATP5J,ATP6AP2,ATP6V1C1,ATP7B,ATR,ATRX,AURKA, AUTS2,AVEN,AXIN2,AZGP1,B3GALNT1,B3GNT3,B3GNT7,B4GALNT2,BACE1,BACH1,BAD, BAG1,BAG2,BAG3,BAIAP2L1,BAMBI,BARD1,BARX2,BATF2,BAX,BAZ2A,BCAM,BCAR1, BCAR3,BCAS2,BCAS3,BCAT1,BCL10,BCL2,BCL2L12,BCL2L2,BCL3,BCL6,BCL9,BCL9L, BCOR,BCORL1,BCR,BDKRB2,BDNF,BET1,BHMT,BID,BIK,BIN1,BIRC3,BIRC5,BIRC6, BLCAP,BLID,BMF,BMP1,BMP10,BMP3,BMP4,BMP6,BMP7,BMPER,BMPR1A,BNC1,BNIP3L, BOLL,BOP1,BPI,BPTF,BRCA1,BRCA2,BRCC3,BRD4,BRD8,BRE,BRF2,BRIP1,BRMS1, BRMS1L,BRSK2,BST2,BTBD7,BTC,BTG1,BTG2,BTG3,BTK,BTLA,BTRC,BUB1,BUB1B,BVES,C10orf10,C19orf48,C1orf61,C2orf40,C3,C5,C6,C6orf106,C7,C8orf4,C9,CA2,CA9,CACNA2D3,CACYBP,CAD,CADM1,CALU,CAMK2N1,CAMP,CAMSAP1,CANT1,CAPZA1,CARD10,CARM1,CARS,CASK,CASP4,CASP6,CASP8,CASR,CASS4,CAV1,CBL,CBX2,CBX4,CBX5,CBX7, CBX8,CCDC34,CDC6,CCDC67,CCDC8,CCL1,CCL11,CCL13,CCL14,CCL17,CCL18,CCL19, CCL2,CCL20,CCL21,CCL22,CCL4,CCL5,CCL7,CCL8,CCNA2,CCNB1,CCNE1,CCNG2, CCNH,CCNY,CCR1,CCR3,CCR4,CCR6,CCR7,CCR9,CCRL2,CCT2,CD109,CD14,CD163, CD163L1,CD164,CD1A,CD1C,CD1D,CD2,CD200,CD226,CD244,CD248,CD27,CD274,CD33, CD36,CD38,CD4,CD40,CD46,CD47,CD48,CD53,CD55,CD58,CD63,CD69,CD70,CD74,CD81,CD82,CD86,CD9,CD93,CD96,CD99,CDA,CDC14A,CDC20,CDC25A,CDC25B,CDC25C, CDC37,CDC42,CDC6,CDC7,CDC73,CDCA5,CDCA7L,CDCA8,CDCP1,CDH1,CDH13,CDH17,CDH2,CDH22,CDK2AP1,CDK3,CDK4,CDK5,CDK5RAP3,CDK6,CDK7,CDK8,CDKN1A, CDKN1C,CDKN2A,CDKN2B,CDKN3,CDT1,CDX1,CDX2,CEACAM1,CEACAM5,CEP55, CEP70,CES2,CFL1,CFTR,CGA,CHAD,CHAT,CHD1,CHD1L,CHD5,CHD8,CHEK1,CHEK2, CHGB,CHI3L1,CHL1,CHML,CHRM3,CHRNA1,CHRNA3,CHST11,CIAPIN1,CIITA,CISD2,CISH,CITED1,CITED2,CIZ1,CKAP2,CKAP4,CKB,CLC,CLCA1,CLCA2,CLCA4,CLDN10,CLDN11,

CLDN16,CLDN3,CLEC3A,CLEC3B,CLEC5A,CLIC4,CLOCK,CLPTM1L,CLU,CMTM3,CMTM8
,
CNN3,CNOT7,CNTF,CNTN1,COIL,COL11A1,COL18A1,COL1A1,COL1A2,COL3A1,COL4A2
,
COL5A1,COL5A2,COL6A1,COMMD1,COMT,CORT,COTL1,COX7A2,CP,CPA4,CPE,CPEB4
,
CPM,CPNE3,CPOX,CPS1,CPSF2,CPT1A,CPZ,CR1,CRABP1,CRCT1,CREB1,CREB3,
CREB3L1,CREB3L2,CREB5,CREBBP,CREBZF,CRH,CRHR1,CRHR2,CRIM1,CRISP3,CRK,
CRKL,CRNN,CRP,CRTC1,CRTC3,CRX,CS,CSE1L,CSF1,CSF2,CSK,CSMD1,CSNK1A1,
CSPG4,CST6,CST7,CTAG2,CTBP1,CTBP2,CTCF,CTCFL,CTGF,CTHRC1,CTLA4,CTNNA1,
CTNNB1,CTNND1,CTTN,CUEDC2,UL4A,CUX1,CX3CL1,CX3CR1,CXCL1,CXCL10,CXCL11
,
CXCL12,CXCL13,CXCL14,CXCL17,CXCL2,CXCL3,CXCL5,CXCL6,CXCL9,CXCR4,CXCR5,
CXCR6,CXXC4,CXXC5,CYB5A,CYB5D2,CYFIP1,CYHR1,CYLD,CYP17A1,CYP19A1,CYP1
A1,
CYP1B1,CYP24A1,CYP26A1,CYP27A1,CYP27B1,CYP2A6,CYP2B6,CYP2C8,CYP2E1,
CYP2J2,CYP2R1,CYP39A1,CYP3A4,CYP3A43,CYP3A5,CYP4F8,CYR61,DAB1,DAB2,
DAB2IP,DACH1,DACH2,DACT1,DACT2,DAND5,DAP,DAP3,DBF4,DBN1,DCC,DCK,DCLK1,
DCN,DCT,DCTN1,DCUN1D1,DCX,DCXR,DDB2,DDC,DDIT3,DDR2,DDT,DDX1,DDX11,
DDX20,DDX27,DDX43,DEDD,DEK,DEPDC1B,DES,DFNA5,DGCR6,DGCR6L,DGCR8,
DHCR24,DHFR,DHRS7,DHX32,DIAPH1,DIAPH3,DICER1,DIO3,DIRAS1,DIRAS3,DIS3,
DIXDC1,DKK1,DKK2,DLC1,DLD,DLEC1,DLG5,DLK1,DLK2,DLL4,DLX2,DLX5,DMGDH,DMP
1,DNAJA1,DNAJB1,DNAJC13,DNM3,DNMT1,DNMT3A,DOCK1,DOCK2,DOCK3,DOCK4,
DOCK5,DOCK8,DOT1L,DPF3,DPH3,DPP4,DPPA2,DPYD,DR1,DRD2,DSC1,DSG1,DSG3,
DTX3L,DUOX2,DUSP1,DUSP4,DUSP6,DYRK1B,DYRK2,E2F1,E2F2,E2F3,E2F4,E2F5,E2F
6,EBAG9,EBF2,EBP,ECD,ECH1,ECM1,ECT2,EDIL3,EDNRA,EDNRB,EEF1A2,EFEMP1,EF
HD2,EFNA1,EFNA3,EFNB2,EGF,EGFL7,EGFR,EGR1,EGR3,EHD1,EHMT1,EI24,EIF3A,EIF
3E,
EIF3H,EIF3I,EIF4E,EIF5A2,EIF6,ELAC2,ELAVL1,ELF3,ELF5,ELMO2,ELP3,ELOVL6,EMILIN
1,EMP2,EMX2,ENAH,ENG,ENO1,ENPP2,ENTPD5,EP300,EPAS1,EPB41L3,EPC1,EPCAM,
EPHA1,EPHA2,EPHA3,EPHA4,EPHA6,EPHA7,EPHA8,EPHB2,EPHB3,EPHB4,EPHB6,EPH
X2,
EPO,EPS15,EPS8,EPSTI1,ERBB3,ERBB4,ERC1,ERCC1,ERCC2,ERCC3,ERCC5,ERG,
ERGIC1,ERGIC3,ERP29,ESD,ESPL1,ESPN,ESR1,ESR2,ESRP1,ETS1,ETV1,ETV4,ETV5,
ETV6,EVI5,EVL,EVX1,EWSR1,EXO1,EXT1,EXTL3,EYA1,EYA2,EYA4,EZH2,EZR,F10,F11,F
2,F5,F8,F9,FABP1,FABP4,FABP5,FABP7,FADD,FAF1,FAIM2,FAM107A,FAM120A,FAM129
B,
FAM134B,FAM20C,FAM3B,FAM3C,FAM83D,FANCA,FANCC,FANCD2,FANCF,FAP,FAS,
FASN,FASTKD2,FAT4,FBN1,FBN2,FBXL5,FBXO11,FBXO32,FBXO4,FBXO45,FBXW7,FCN
2,FDPS,FECH,FEM1A,FER,FEV,FEZ1,FEZF1,FGD4,FGF1,FGF10,FGF14,FGF18,FGF2,FG
F23,FGF3,FGF4,FGF5,FGF7,FGF8,FGF9,FGFBP1,FGFR1,FGFR2,FGFR3,FGFR4,FGG,FG
R,FH,
FHIT,FHL1,FHL2,FHOD1,FHOD3,FJX1,FKBP14,FKBPL,FLI1,FLNA,FLOT2,FLT1,FLT3,FLT4
,
FMNL2,FMNL3,FN1,FNDC3B,FOSB,FOXA1,FOXA2,FOXC1,FOXC2,FOXD3,FOXE1,FOXF1
,
FOXF2,FOXG1,FOXH1,FOXJ1,FOXJ3,FOXL1,FOXL2,FOXM1,FOXO1,FOXO3,FOXO4,
FOXP1,FOXP2,FOXP3,FOXQ1,FOXR2,FRAS1,FRAT1,FRMD4A,FRY,FRYL,FSCN1,FSHR,
FST,FSTL1,FURIN,FUS,FUT3,FUT4,FUT5,FUT8,FXYD3,FXYD5,FXYD6,FYN,FZD1,FZD2,
FZD5,FZD8,G3BP1,GAB1,GAB2,GABARAPL1,GABRP,GADD45A,GADD45G,GAK,GAL,
GAL3ST2,GALC,GALNT14,GALNT2,GALNT3,GALNT9,GAN,GAS1,GAS6,GAS8,GAST,

GATA2,GATA4,GATA5,GATA6,GBP1,GBP2,GBX2,GC,GCG,GCKR,GDF15,GDF2,GDF3,
GDNF,GEM,GEMIN5,GFAP,GFI1,GGH,GHRH,GIP,GIPC1,GIT1,GJA3,GJB5,GKN1,GLA,GL
B1,GLE1,GLI1,GLI2,GLIPR1,GLO1,GMDS,GNA13,GNA15,GNAI2,GNAI3,GNAQ,GNAS,GNE
,
GNG2,GNRH1,GOLPH3,GP2,GPC5,GPI,GPNMB,GPR171,GPR18,GPR32,GPR34,GPR39,
GPR55,GPR87,GPRC5A,GPX2,GPX3,GRB14,GRB2,GRHL2,GRHL3,GRIN2A,GRK5,
GRK6,GRM1,GRM4,GRM5,GRPR,GSC,GSN,GSPT1,GSTM3,GSTP1,GTSE1,GUCY2C,
GUK1,H3F3A,HABP2,HACE1,HADHA,HAO2,HAPLN3,HAS1,HAS2,HAX1,HBD,HBEGF,HBP
1,HCCS,HDAC1,HDAC2,HDAC4,HDAC6,HDAC7,HDAC8,HDGF,HECTD1,HECTD2,HELLS,
HEPACAM,HERC5,HES1,HES5,HEXA,HEXIM1,HEY1,HFE,HGS,HHIP,HIF1A,HINT1,HIP1,
HIPK1,HIPK2,HIRA,HJURP,HK1,HK3,HLTF,HMGA1,HMGA2,HMGB3,HMGCR,HMGCS2,
HMGN1,HMGN5,HMMR,HN1,HNF1A,HNF4A,HNRNPA2B1,HOMER1,HOOK1,HOPX,HOXA
1,HOXA11,HOXA13,HOXA5,HOXA9,HOXB2,HOXB9,HOXC11,HPD,HPGD,HPR,HPSE,HR,
HRG,HS3ST2,HSD17B2,HSD3B1,HSF1,HSP90B1,HSPA14,HSPA2,HSPA9,HSPB8,HTATIP
2,HTRA1,HTRA2,HTRA3,HTT,HUNK,HUWE1,HYOU1,IAPP,ICMT,ID1,ID2,ID3,ID4,IDH1,IDH
2,
IDO1,IER2,IFI27,IFIT2,IFIT3,IFNG,IGF1R,IGF2,IGF2BP1,IGF2BP2,IGF2BP3,IGFBP1,IGFBP
2,
IGFBP3,IGFBP5,IGFBP7,IGFBPL1,IKBKB,IKZF1,IL10,IL13,IL17A,IL17F,IL18,IL1A,IL1R1
,IL23R,IL24,IL33,IL4,IL6R,IL7,ILK,IMP3,IMPACT,INA,ING1,ING2,ING3,ING5,INHBA,INSIG2,
INSM1,IQGAP1,IQGAP2,IRAK1,IRF8,IRX2,IRX5,ITGA2,ITGA3,ITGA5,ITGA7,ITGA8,ITGA9,
ITGAV,ITGB1,ITGB3,ITGB4,ITGB8,ITGBL1,ITIH5,JAG1,JAG2,JAK1,JAK2,JAK3,JAZF1,
JMJD6,JUN,KAT2A,KCNJ1,KCNK9,KCNMA1,KCNQ1,KCNRG,KDM1A,KDM2A,KDM3A,
KDM4A,KDM4B,KDM4C,KDM5B,KDM5C,KDM6A,KDR,KEAP1,KIAA0101,KIF11,KIF14,KIF1
5,KIF18A,KIF1B,KIF26B,KIF2A,KIF3A,KIF3C,KIF5B,KIFC1,KIN,KIR2DL4,KISS1,KIT,KITLG,
KL,
KLB,KLF12,KLF15,KLF17,KLF2,KLF4,KLF5,KLF6,KLHDC4,KLK10,KLK14,KLK15,KLK3,KLK
7,KLK8,KLRG1,KMO,KPNA2,KPNB1,KRAS,KRT19,KRT20,KRT7,KY,LAMA4,LAMA5,LAMB1
,
LAMB3,LAMB4,LAMC1,LAMC2,LAMP1,LAMP2,LAMP3,LAP3,LAPTM4B,LARP1,LARP7,LAR
S,LASP1,LAT,LATS1,LATS2,LBH,LBX1,LCK,LCT,LDB1,LEMD3,LEP,LEPREL1,LETM1,LFN
G,
LGALS3,LGALS3BP,LGALS9,LGI4,LGR5,LHCGR,LIF,LIFR,LIG4,LILRB1,LILRB2,LIMD2,
LIMK2,LIN28B,LMNA,LMO2,LMO4,LMO7,LMX1B,LOX,LOXL2,LPAR1,LPCAT1,LPHN3,LPIN
2,LPP,LRG1,LRP1,LRP1B,LRP5,LRP6,LRP8,LRPPRC,LRRC3B,LRRC4,LRRFIP1,LSAMP,
LSM1,LSP1,LTBP4,LXN,LY75,LYAR,LYN,LZTFL1,LZTS1,MACROD2,MADD,MAGEA1,
MAGEC2,MAGED4B,MAGI1,MAK,MAL,MAL2,MALL,MALT1,MAML1,MAML2,MAOA,MAP1B,
MAP1S,MAP2,MAP2K1,MAP2K4,MAP3K1,MAP3K2,MAP3K7,MAP3K8,MAP3K9,MAP4K3,
MAP4K4,MAPK1,MAPK8,MAPKAPK2,MAPT,MARCKS,MARCO,MARK4,MARVELD3,MAS1,
MAT2A,MAT2B,MAX,MB,MBD1,MBD2,MBD4,MBP,MCC,MCM2,MCM4,MCRS1,MDM4,ME1,
MED1,MED12,MED19,MED27,MEF2C,MEF2D,MEIS1,MEMO1,MEN1,MEST,MET,METTL13
,
MFAP3L,MFSD2A,MGA,MGAT1,MGAT3,MGMT,MGST1,MIA,MIB1,MICAL2,MIF,MINA,MIP,
MITF,MKL1,MLF2,MLN,MME,MMP10,MMP11,MMP12,MMP13,MMP14,MMP16,MMP28,
MMP7,MMP9,MNT,MOBP,MOS,MOV10,MPI,MPL,MPO,MPZL1,MRC2,MSC,MSH3,MSH6,
MSMB,MSN,MSRA,MST1R,MSX2,MT3,MT4,MTA1,MTA2,MTA3,MTAP,MTBP,MTDH,
MTHFD2,MTHFR,MTMR3,MTOR,MTRR,MTSS1,MTUS1,MUC13,MUC16,MUC17,MUC2,
MUC20,MUC4,MUC7,MUM1,MUSK,MUT,MUTYH,MVD,MVP,MX1,MX2,MXD1,MXI1,MXRA5
,
MYBL2,MYC,MYCN,MYD88,MYEOV,MYH9,MYL9,MYLK,MYO5B,MYO9B,MZF1,NAB2,

NAMPT,NAP1L1,NAT1,NAT2,NAV1,NBN,NCALD,NCK1,NCK2,NCL,NCOA1,NCOA2,NCOA3
,
NCOA5,NCOR1,NCOR2,NCSTN,NDC80,NDP,NDRG2,NDRG3,NDRG4,NDUFB6,NDUFB9,
NDUFS6,NEB,NEDD1,NEDD4,NEDD4L,NEDD8,NEFL,NEIL1,NEIL2,NEK2,NEK6,NEK7,NES
,
NET1,NETO2,NEU3,NEUROD1,NF1,NFAT5,NFATC2,NFIB,NFKB1,NFKBIA,NGF,NGFR,NH
S,NIPSNAP1,NISCH,NKD1,NKD2,NKTR,NLK,NLRP1,NLRP3,NME1,NME4,NME6,NMI,NMU,
NNAT,NNT,NOB1,NOD1,NOD2,NODAL,NOG,NOP14,NOS1,NOS2,NOS3,NOTCH1,NOTCH
3,NOTCH4,NOV,NOX1,NOX4,NPAS2,NPC1,NPL,NPM1,NPS,NPY,NPY1R,NQO2,NR0B1,
NR1D1,NR1I2,NR1I3,NR2F1,NR2F2,NR3C2,NR4A2,NR4A3,NR5A2,NR6A1,NRAS,NRK,NR
L,NT5E,NTRK1,NTRK3,NTS,NTSR1,NUAK1,NUCB2,NUCKS1,NUMB,NUP88,NUSAP1,OAT
,
ODAM,ODF4,OLA1,OLFM4,OLIG1,OLIG2,ONECUT2,ORAI1,ORAOV1,OSM,OSMR,OTC,OT
P,OTUB1,OTUD3,P4HA1,P4HA2,PABPC1,PACRG,PADI4,PAFAH1B1,PAH,PAK2,PAK3,PA
K4
,PAK6,PALB2,PAM,PARD3,PARG,PARP1,PARVA,PARVB,PAX3,PAX4,PAX5,PAX6,PAX7,
PAX8,PBK,PBRM1,PBX3,PC,PCBP1,PCBP2,PCDH10,PCDH9,PCK2,PCNA,PDC,PDCD4,
PDCD5,PDCD6,PDCL3,PDF,PDGFB,PDGFRA,PDGFRB,PDIA3,PDK3,PDLIM2,PDLIM5,
PDPN,PDSS2,PDX1,PEBP1,PEG10,PELP1,PER1,PER2,PERP,PFKFB2,PFKFB3,PFN2,PG
C,PGF,PGK1,PGRMC2,PHF10,PHF20,PHF8,PHIP,PHLDA1,PHLPP1,PHLPP2,PHOX2B,PIA
S1,PICK1,PIGF,PIGR,PIGS,PIEZO1,PIK3C2G,PIK3CA,PIK3CB,PIK3CD,PIK3CG,PIK3R1,PI
K3R3,PIKFYVE,PIN1,PINK1,PIP4K2B,PIR,PITPNC1,PITX1,PITX2,PIWIL1,PIWIL2,PIWIL4,P
KN1,
PKP1,PKP2,PLA2G16,PLA2G7,PLAC1,PLAG1,PLAGL1,PLAU,PLAUR,PLCE1,PLCG1,PLD2
,
PLEKHA5,PLK1,PLOD2,PLS3,PLXDC1,PLXNA2,PMAIP1,PMEPA1,PML,PMP22,PMS2,
PNLIPRP3,POLB,POLE,POLI,POLR2A,POMC,PON1,POSTN,POT1,POU2F1,POU3F2,
POU3F3,PPA1,PPARG,PPM1A,PPM1B,PPM1D,PPM1F,PPM1H,PPP1CA,PPP1R13L,
PPP1R3B,PPP2CA,PPP2R2C,PRAME,PRC1,PRDM10,PRDM14,PRDM5,PRDX1,PRDX4,
PRDX6,PREP,PAG1,PTK2B,PREX2,PRICKLE1,PRKAA1,PRKAR1A,PRKCD,PRKCDBP,
PRKCI,PRKCZ,PRKD1,PRKD2,PRKX,PRL,PRMT1,PRMT2,PRMT5,PRMT6,PRMT7,PRNP,
PROK1,PROM1,PROX1,PRR15,PRSS3,PRUNE,PRUNE2,PRX,PSAT1,PSCA,PTBP2,PTCH
1,PTEN,PTER,PTGIS,PTGS2,PTH,PTH1R,PTHLH,PTK6,PTK7,PTOV1,PTP4A1,PTP4A3,
PTPN12,PTPN13,PTPN14,PTPN4,PTPRF,PTPRG,PTPRT,PTS,PTTG1IP,PTX3,PVR,PYY,
QKI,QRFP,RAB11A,RAB14,RAB17,RAB1A,RAB22A,RAB25,RAB27B,RAB32,RAB3D,RAB4
0B,RAB40C,RAB5C,RABL3,RAC1,RAC2,RACGAP1,RAD18,RAD21,RAD50,RAD51,RAD52,
RAD54B,RAF1,RAI2,RALA,RALB,RALBP1,RALY,RAMP3,RAN,RANGAP1,RAP1A,RAP1B,
RAP1GAP,RAP2A,RAP2B,RAPGEF2,RAPH1,RARB,RARRES1,RASA1,RASAL1,RASAL2,
RASGRF1,RASGRF2,RASGRP1,RASGRP3,RASSF1,RASSF3,RASSF7,RASSF8,RB1,
RB1CC1,RBBP4,RBBP6,RBBP8,RBM3,RBM47,RBM5,RBP1,RBP2,RBX1,RCAN3,RD3,REG
4,RELA,REPS2,RERG,RET,REV3L,RFC1,RFX6,RFXAP,RGMB,RGS1,RGS16,RGS17,RGS
2,
RGS22,RGS6,RGSL1,RHBDD2,RHO,RHOB,RHOBTB2,RHOC,RHOD,RHOG,RHOJ,RHOT1
,
RHOU,RIN1,RIOK3,RIPK1,RIPK2,RIPK3,RLF,RNASEL,RND3,RNF111,RNF13,RNF180,RNF
2,RNF4,RNF40,RNF43,RNH1,ROBO1,ROBO3,ROBO4,ROCK1,ROCK2,ROR1,ROR2,RORA
,
RP1,RPA1,RPE,RPL15,RPL26,RPL39,RPL41,RPL7,RPN2,RPS12,RPS3,RPS6KB1,RPSA,
RRBP1,RRM1,RRM2,RRP1B,RSPO2,RTKN,RUFY3,RUNX1,RUNX1T1,RUNX2,RUNX3,
RXFP1,RXRA,RYBP,S100A11,S100A2,S100A6,S100A7,S100A8,S100A9,S100B,S100P,
S100PBP,S100Z,S1PR3,SAA2,SACS,SALL4,SAMD9,SARS,SART1,SART3,SASH1,SATB2,

SCAI,SCAMP1,SCARA5,SCGB2A2,SCN7A,SCRIB,SCUBE2,SCUBE3,SDC1,SDC2,SDC4,
SDCBP,SDHA,SDHC,SDPR,SEC14L2,SEC23A,SEC62,SEL1L,SELP,SEMA3E,SEMA3F,SE
MA4C,SEMA6D,SENP1,SENP2,SENP3,SERPINA1,SERPINA3,SERPINA5,SERPINB13,
SERPINB2,SERPINB3,SERPINB5,SETD2,SETDB1,SF3B1,SFPQ,SFRP1,SFRP2,SFRP4,
SGPP1,SGPP2,SGTA,SH2B1,SH3BGRL,SH3GL2,SH3GLB2,SH3PXD2B,SHB,SHE,SHH,
SHISA3,SHMT1,SHOC2,SHOX2,SI,SIAH2,SIN3A,SIPA1,SIRT1,SIRT2,SIRT3,SIX2,SIX3,SIX
4,SKA3,SKAP2,SKI,SKP1,SKP2,SLC19A1,SLC19A3,SLC22A17,SLC22A18,SLC25A1,SLC2
8A3,
SLC29A1,SLC29A3,SLC2A1,SLC38A1,SLC39A14,SLC5A8,SLC6A14,SLC7A11,SLC7A5,
SLCO1B1,SLCO1B3,SLIT3,SLITRK3,SLITRK6,SLK,SLN,SMAD1,SMAD2,SMAD3,SMAD4,
SMAD5,SMAD7,SMAP1,SMARCA4,SMARCA5,SMARCB1,SMARCC1,SMARCD1,SMC4,SM
O,SMR3A,SMS,SMURF1,SMURF2,SMYD2,SMYD3,SNAI2,SNAI3,SNAPIN,SND1,SNTB2,S
NX6,SNX9,SOCS1,SOCS2,SOCS3,SOD2,SOD3,SOHLH2,SOS1,SOST,SOX10,SOX11,SOX
12,
SOX17,SOX2,SOX3,SOX4,SOX6,SOX7,SOX8,SOX9,SP1,SP100,SP3,SP6,SPAG5,SPAG9,
SPARC,SPARCL1,SPDEF,SPIN1,SPINT2,SPOCK1,SPOP,SPR,SPRED1,SPRR2A,SPRR3,
SPRY1,SPRY2,SPRY4,SPTAN1,SQLE,SQSTM1,SRC,SRD5A1,SRD5A2,SRF,SRPK1,SRR
M4,
SSBP1,SSRP1,SST,SSTR2,SSTR3,SSTR5,SSX1,SSX2IP,ST13,SLC9A9,ST3GAL6,
ST6GALNAC2,STAG2,STARD10,STARD13,STAT1,STAT3,STAT4,STAT5B,STAT6,STC2,S
TEAP1,STEAP2,STIM2,STIP1,STK33,STK39,STK4,STMN1,STRAP,STRN,STS,STX6,STYK
1,
SUDS3,SUFU,SULF1,SULF2,SULT1E1,SUMO1,SUMO3,SUSD2,SUZ12,SVEP1,SYK,TAC1,
TACC2,TACC3,TACSTD2,TAGLN,TAGLN2,TANK,TARBP2,TAT,TBC1D16,TBK1,TBP,TBX2,
TBX21,TBX3,TBX4,TBX5,TBXAS1,TCEB1,TCF12,TCF21,TCF3,TCF4,TCF7,TCF7L2,TDGF1
,
TDO2,TDP1,TDRD1,TEAD4,TEC,TEF,TEK,TERT,TES,TET1,TET2,TEX101,TF,TFAP2A,
TFAP2C,TFAP2E,TFCP2,TFEB,TFF1,TFF2,TFF3,TFG,TFPI,TFPI2,TG,TGFA,TGFB1,TGFBI,
TGFBR2,TGFBR3,TGIF1,TGM2,TGM3,THBS1,THBS4,THOC1,THRSP,THY1,TIAF1,TIAM2,
TIMELESS,TIMM17A,TIMP2,TIMP3,TIMP4,TJP1,TKTL1,TLE1,TLE4,TLN1,TLR3,TLR4,TLR5
,
TLR7,TLR8,TLR9,TM4SF1,TM4SF5,TM9SF4,TMED3,TMEM100,TMEM127,TMEM14A,
TMEM174,TMEM207,TMEM25,TMEM33,TMEM45B,TMEM97,TMOD1,TMPRSS2,TMPRSS3
,
TMPRSS4,TNFAIP1,TNFRSF10A,TNFRSF10B,TNFRSF1B,TNK2,TNS1,TNS3,TNS4,TOB1,
TOB2,TOM1,TOM1L1,TOMM34,TOP1,TOPBP1,TOX,TP53,TP53BP2,TP53INP1,TP63,TP73,
TPD52,TPM3,TPO,TPR,TRAF2,TRAF4,TRAF6,TREM2,TRH,TRIB1,TRIB2,TRIB3,TRIM16,
TRIM31,TRIM33,TRIM37,TRIM44,TRIM59,TRIM66,TRIM9,TRIT1,TRPA1,TRPC1,TRPC6,
TRPM2,TRPM7,TRPM8,TRPV1,TRPV5,TRPV6,TSC1,TSC2,TSG101,TSLP,TSPAN1,
TSPAN12,TSPAN7,TSPAN8,TSPAN9,TSPYL5,TSSC1,TTC4,TTF1,TTK,TTYH2,TUSC1,
TWIST1,TXN,TFE3,TRPS1,TUBB3,TUSC3,TYK2,TYMS,TYR,TYRO3,TYRP1,U2AF2,UBE2C
,
UBE2D3,UBE2Q1,UBIAD1,UCHL1,UCN,UGT2B17,ULBP2,UNC5B,UPF1,USF1,USP10,
USP14,USP15,USP18,USP22,USP28,USP37,USP4,USP9X,UVRAG,VANGL2,VAPB,VASP,
VAV2,VAV3,VCAM1,VCP,VDAC1,VDR,VEGFC,VEZT,VGLL2,VHL,VIM,VIP,VPS25,VRK1,
VTCN1,VTI1A,VWF,WASF2,WBSCR22,WDR26,WEE1,WIF1,WIPI1,WIPI2,WISP1,WISP2,WI
SP3,WNT10A,WNT10B,WNT11,WNT2,WNT3,WNT3A,WNT4,WNT5B,WNT6,WNT7A,WRN,
WT1,WTAP,WWP1,WWP2,WWTR1,XAF1,XBP1,XCR1,XG,XPA,XPO1,XPO5,XPO6,YBX1,
YTHDC2,YWHAG,YWHAH,YWHAZ,YY1,ZAP70,ZBED3,ZBTB16,ZBTB20,ZBTB7A,ZBTB8A,
ZEB2,ZFX,ZIC1,ZKSCAN3,ZMAT1,ZNF165,ZNF217,ZNF281,ZNF300,ZNF304,ZNF331,ZNF
395,ZNF419,ZNF444,ZNF488,WWOX

S5B Table. Genes in gene set for metastasis function
ABCB1,ABCB5,ABCC3,ABCE1,ABCG2,ABL2,ABR,ACE2,ADAM10,ADAM19,ADAM8,ADAM9,ADAMTS1,<br>ADAMTS5,ADIPOR1,ADIPOR2,ADM,ADO,ADORA2B,ADRB2,AFAP1,AFAP1L1,AFP,AGR2,AHNAK,<br>AIM1,AKAP12,AKAP13,AKR1C3,AKT1,AKT2,AKT3,ALDH1A1,ALDH1A3,ALK,AMOTL2,ANG,ANGPTL2,<br>ANO1,ANO9,ANXA1,ANXA11,ANXA7,APC,APLP2,AQP3,AQP5,AQP9,ARF6,ARID1A,ARID2,ARID3B,<br>ARID4A,ARRDC3,ATAD2,ATF3,ATF4,ATG4A,ATG5,ATM,ATOH1,ATOH8,ATP6AP2,ATP6V1C1,ATR,<br>AURKA,AXIN2,B3GNT7,B4GALNT2,BACE1,BAD,BAG3,BAMBI,BATF2,BAX,BCAR1,BCAS3,BCAT1,<br>BCL2,BCL2L2,BCL3,BCL6,BCL9,BDNFBIK,BIN1,BIRC5,BIRC6,BMP4,BMP7,BOLL,BPTF,BRCA1,<br>BRCA2,BRD4,BRD8,BRMS1,BTBD7,BTG1,BTG2,BTG3,BTK,BVES,C3,C5,C6,C8orf4,C9,CA2,CA9,<br>CACNA2D3,CACYBP,CADM1,CALU,CAMP,CASP6,CBL,CBX7,CBX8,CCDC34,CCL17,CCL18,CCL19,<br>CCL2,CCL20,CCL21,CCL22,CCL5,CCL7,CCNE1,CCNG2,CCR1,CCR4,CCR6,CCR7,CCR9,CD164,CD36,<br>CD38,CD4,CD40,CD47,CD55,CD63,CD70,CD74,CD81,CD82,CD86,CD9,CD99,CDA,CDC20,CDC25A,<br>CDC25B,CDC25C,CDC37,C42,CDCP1,CDH1,CDH17,CDH22,CDK4,CDK5,CDK5RAP3,CDK6,CDK8,<br>CDKN1A,CDKN2A,CDX2,CEACAM1,CEACAM5,CFL1,CHD1L,CHEK1,CHI3L1,CHST11,CIAPIN1,CLCA1,<br>CLCA2,CLDN10,CLOCK,CLU,CMTM3,CNTN1,COIL,COMMD1,CP,CPE,CPT1A,CRCT1,CRHR2,CRK,<br>CRKL,CRP,CS,CSE1L,CSF1,CSK,CSPG4,CST6,CTBP1,CTBP2,CTGF,CTHRC1,CTLA4,CTTN,CUL4A,<br>CUX1,CX3CR1,CXCL1,CXCL10,CXCL12,CXCL13,CXCL14,CXCL17,CXCL3,CXCL5,CXCL6,CXCL9,<br>CXCR4,CXCR5,CXCR6,CYB5D2,CYLD,CYP1B1,CYP2E1,CYP2J2,CYP3A4,CYR61,DAB2IP,DACT2,<br>DAND5,DAP,DCC,DCLK1,DDR2,DDX11,DDX20,DEK,DES,DHRS7,DKK1,DKK2,DLL4,DNM3,DNMT1,<br>DNMT3A,DOCK1,DOT1L,DPH3,DPP4,DUSP1,DUSP6,DYRK1B,DYRK2,E2F1,E2F3,ECD,ECM1,ECT2,<br>EDIL3,EEF1A2,EFEMP1,EGF,EGFL7,EGFR,EGR1,EIF3I,EIF4E,EIF5A2,EIF6,EMILIN1,EMP2,ENAH,<br>ENG,ENO1,EPB41L3,EPCAM,EPHA2,EPHB2,EPHB4,EPHB6,ERBB3,ERBB4,ERCC1,ERCC2,ERCC3,<br>ERG,ERGIC1,ESR1,ETS1,ETV1,EXT1,EYA2,EZH2,F11,F5,F8,FABP7,FADD,FANCA,FANCC,FANCD2,<br>FAP,FAS,FASTKD2,FAT4,FBXL5,FBXO11,FBXW7,FGF10,FGF18,FGF2,FGFR1,FGFR2,FGFR3,FGFR4,<br>FH,FHIT,FHL1,FHL2,FJX1,FLI1,FLNA,FLOT2,FLT1,FN1,FOSB,FOXA1,FOXA2,FOXC1,FOXC2,FOXD3,<br>FOXL1,FOXM1,FOXO1,FOXO3,FOXO4,FOXP1,FOXP2,FOXP3,FOXQ1,FRAT1,FSCN1,FSHR,FURIN,<br>FUS,FUT3,FUT4,FYN,FZD5,FZD8,GAB1,GAB2,GADD45A,GAL,GALNT14,GALNT2,GAS6,GATA6,GC,<br>GDNF,GEMIN5,GHRH,GJA3,GKN1,GLB1,GLI1,GLI2,GLIPR1,GLO1,GNA13,GNAQ,GOLPH3,GPC5,GPI,<br>GPNMB,GPR171,GPR39,GPR55,GPR87,GPX3,GRB14,GRB2,GRK6,GRM1,HABP2,HAS2,HBD,HBP1,<br>HDAC1,HDAC2,HDAC4,HDAC6,HES1,HES5,HEXA,HIF1A,HIPK2,HJURP,HMGA1,HMGA2,HMGB3,<br>HMMR,HNRNPA2B1,HOOK1,HOXA1,HOXA5,HOXA9,HOXB2,HOXB9,HPR,HRG,HS3ST2,HSPA14,<br>HTATIP2,HTRA1,HYOU1,ICMT,ID1,ID2,ID3,IDH2,IFIT2,IGF1R,IGF2,IGF2BP1,IGFBP1,IGFBP3,IGFBP5,<br>IGFBP7,IL13,ILK,IMP3,IMPACT,ING1,ING2,ING3,ING5,IQGAP1,IRAK1,IRX2,ITGA2,ITGA3,ITGA5,ITGAV,<br>ITGB1,ITGB3,JAG1,JAG2,JAK1,JAK2,JAK3,JMJD6,JUN,KCNJ1,KCNMA1,KDM2A,KDM4B,KDM5B,KDR,<br>KEAP1,KIF11,KIF14,KIF15,KIF2A,KIF3C,KISS1,KIT,KITLG,KL,KLF17,KLF2,KLF4,KLF5,KLF6,KPNA2,<br>KRAS,KRT19,KRT7,LAMB3,LAMC2,LAMP1,LAMP3,LAP3,LAPTM4B,LASP1,LATS1,LATS2,LIF,LIMK2,<br>LIN28B,LMX1B,LOX,LOXL2,LRP1,LRP1B,LRP5,LRPPRC,LRRC4,LSM1,LYN,LZTS1,MACROD2,MADD,<br>MAP2K4,MAP3K2,MAP3K9,MAP4K4,MAPK1,MAPKAPK2,MARCKS,MAX,MB,MBD2,MBP,MCM2,MDM4,<br>MED1,MED12,MED27,MEF2C,MEIS1,MET,MFAP3L,MGAT1,MGMT,MIA,MIB1,MIF,MIP,MITF,MKL1,<br>MLF2,MME,MMP10,MMP12,MMP13,MMP14,MMP28,MMP7,MMP9,MSC,MT3,MTA1,MTA2,MTA3,MTBP,<br>MTDH,MTMR3,MTOR,MTSS1,MTUS1,MUC16,MUC4,MUSK,MVD,MVP,MX1,MYC,MYCN,MYD88,MYH9,<br>MYO5B,NAP1L1,NAV1,NCK2,NCOA1,NDC80,NDRG2,NEDD1,NEDD4,NEDD4L,NET1,NF1,NFAT5,NGF,<br>NHS,NKD1,NKD2,NLK,NLRP3,NME1,NOB1,NODAL,NOG,NOTCH1,NOTCH3,NOV,NOX1,NOX4,NPM1,<br>NPS,NTSR1,NUAK1,NUMB,NUP88,OLA1,ORAI1,OSM,OTUB1,P4HA1,PAK2,PAK4,PALB2,PARP1,<br>PAX3,PAX7,PAX8,PBK,PC,PCBP1,PCNA,PDC,PDCD4,PDCD5,PDGFRA,PDK3,PDPN,PEG10,PELP1,<br>PER2,PFKFB3,PGC,PHF8,PHLDA1,PIAS1,PICK1,PIGS,PIK3CA,PIKFYVE,PIN1,PIP4K2B,PIR,PIWIL1,<br>PIWIL2,PKN1,PLA2G16,PLAUR,PLD2,PLK1,PMAIP1,PMEPA1,PMP22,POLB,PPARG,PPP1CA,PRAME,<br>PRC1,PRDM14,PREX2,PRKCZ,PRKD1,PRL,PRMT1,PRMT5,PROK1,PROX1,PRSS3,PRUNE,PSCA,<br>PTEN,PTER,PTGS2,PTH,PTK6,PTK7,PTP4A3,PTPN13,PVR,QKI,RAB17,RAB22A,RAB25,RAB27B,

RAB3D,RAB40B,RAB40C,RABL3,RAC1,RAC2,RACGAP1,RALA,RALB,RAMP3,RAN,RAP1A,RAP1B, RAP1GAP,RAP2B,RASAL2,RASSF3,RB1,RBP2,RBX1,RCAN3,RET,RGMB,RGS16,RHBDD2,RHO, RHOB,RHOBTB2,RHOC,RHOJ,RIN1,RIPK1,RIPK3,RNF111,RNF43,ROBO1,ROBO3,ROCK1,ROCK2, ROR1,ROR2,RPA1,RPL39,RPS12,RPS3,RPS6KB1,RREB1,RRM1,RRM2,RRP1B,RSPO2,RUNX2,RUNX3, RXFP1,S100A11,S100A2,S100A6,S100A7,S100A8,S100A9,S100B,S100P,S1PR3,SALL4,SART1,SASH1, SATB2,SCAI,SCAMP1,SCRIB,SDC1,SERPINB2,SETDB1,SFRP1,SFRP2,SH3GL2,SHH,SHMT1,SI,SIAH2, SIRT1,SIRT2,SKI,SKP2,SLC19A3,SLC38A1,SMAD1,SMAD2,SMAD3,SMAD4,SMAD7,SMO,SMYD3, SNAI2,SND1,SNTB2,SOCS3,SOD2,SOD3,SOX10,SOX12,SOX17,SOX2,SOX4,SOX6,SOX7,SOX9,SP1, SP3,SPAG5,SPAG9,SPARC,SPARCL1,SPDEF,SPOCK1,SPOP,SPRY1,SPRY4,SPTAN1,SQLE,SQSTM1, SRC,SRD5A2,SRF,SRPK1,SRRM4,SSTR2,SSX2IP,STARD13,STAT1,STAT3,STAT5B,STIP1,STMN1, STS,SULF1,SULF2,SUZ12,SYK,TACC3,TAGLN,TAT,TBK1,TBX2,TCEB1,TCF4,TCF7,TCF7L2,TDGF1, TEAD4,TERT,TET1,TF,TFF1,TFF3,TFPI,TG,TGFB1,TGFBI,TGIF1,TGM2,THY1,TIMM17A,TIMP2,TKTL1, TLR3,TLR4,TLR9,TM4SF1,TMED3,TMPRSS2,TMPRSS4,TNFAIP1,TNK2,TOB1,TOP1,TOPBP1,TP53, TRAF2,TRAF4,TRAF6,TRIB3,TRIM59,TRPA1,TRPM7,TRPM8,TRPV6,TSC2,TSG101,TSPAN12,TSPAN8, TSPAN9,TTYH2,TWIST1,TXN,TYR,UBE2Q1,UCHL1,UCN,UNC5B,USP10,USP15,USP18,USP22,USP37, USP9X,UVRAG,VASP,VAV2,VAV3,VCP,VEGFC,VEZT,VHL,VIP,VRK1,VTCN1,WBSCR22,WDR26, WEE1,WISP1,WNT10A,WNT10B,WNT3A,WNT5B,WT1,WTAP,WWP1,XAF1,XCR1,XPO1,YWHAZ, YY1,ZBED3,ZBTB7A,ZEB2,ZFX,ZKSCAN3,ZNF217,ZNF300,ZNF304,ZNF488,ALCAM,AR,ASAP1,DACH1, DLC1,PIEZO1,MMP16,PAG1,TRPS1,TUSC3,WWOX

**S5C Table. Genes in gene set for biomarker of metastasis**
ABAT,ABCA13,ABCB1,ABCC2,ABCG2,ACE2,ACP1,ACTN4,ADAM10,ADAM8,ADAM9, ADAMTS1,ADAMTS5,ADO,AFAP1,AFP,AGBL2,AGR2,AIM1,AKR1B10,AKT1,AKT2,ALDH1A1,ALDH1A3,ALK,ALCAM,ANG,ANLN,ANXA1,ANXA2,APC,APOA1,APOBEC3G,AQP1,AQP3, AQP5,AR,ARG1,ARHGEF7,ARID1A,ARPC1B,ASAP1,ASCL2,ASPM,ATAD2,ATF3,ATF5,ATM,ATOH8,BAD,BAMBI,BARX2,BATF2,BAX,BCL10,BCL2,BCL2L12,BCL6,BCOR,BCORL1,BDNF,BID,BIRC5,BMP4,BMP7,BMPR1A,BRCA1,BRCA2,BRE,BRMS1,BTBD7,BTC,BTG1,BTG3, BVES,C10orf10,C3,C6orf106,CA9,CACYBP,CAD,CADM1,CAMP,CASK,CASP8,CASR,CBX7, CCL17,CCL19,CCL2,CCL20,CCL21,CCL4,CCL5,CCL7,CCNB1,CCNE1,CCNG2,CCNY,CCR6,CCR7,CCT2,CD109,CD14,CD163,CD1A,CD4,CD40,CD47,CD55,CD74,CD82,CD9,CDC20, CDC25B,CDC25C,CDC42,CDC6,CDCP1,CDH1,CDH13,CDK4,CDK5,CDK8,CDKN2A,CDX2, CEACAM1,CES2,CFL1,CHD1L,CHEK1,CHEK2,CHRM3,CHRNA3,CIAPIN1,CISH,CLDN16, CLIC4,CLOCK,CLPTM1L,COIL,COL1A1,COL1A2,COMT,CP,CPE,CRK,CRKL,CRNN,CRP, CRTC1,CRTC3,CSE1L,CSNK1A1,CSPG4,CTBP2,CTGF,CTHRC1,CTNNB1,CTTN,CX3CL1, CX3CR1,CXCL1,CXCL10,CXCL12,CXCL14,CXCL5,CXCL9,CXCR4,CXCR6,CYB5A,CYP17A1,CYP19A1,CYP1A1,CYP24A1,CYP2B6,CYP3A4,CYP3A5,CYR61,DAB2,DACH1,DACH2, DACT2,DAND5,DAP,DCC,DCX,DDC,DDX1,DEC1,DEK,DES,DICER1,DIXDC1,DLC1,DLG5, DLK1,DLL4,DLX2,DLX5,DPP4,E2F1,EBP,ECD,ECM1,ECT2,EDIL3,EEF1A2,EFEMP1,EGF, EGR3,EGFL7,EGFR,EIF3E,EIF3I,EIF4E,EIF5A2,ENO1,EPAS1,EPCAM,EPHA2,EPHA3, EPHA7,EPHB4,EPHB6,EPO,EPS15,EPS8,ERBB3,ERBB4,ERCC1,ERG,ERP29,ESR1,ESR2, EYA2,EZH2,F2,FABP1,FABP4,FADD,FAM3C,FANCF,FAP,FAS,FBXO11,FBXW7,FER,FGF14,FGF2,FGFBP1,FGFR1,FGFR2,FGFR3,FGFR4,FHIT,FHL1,FHL2,FKBPL,FLNA,FLT1,FLT3, FLT4,FN1,FOXA1,FOXC1,FOXC2,FOXD3,FOXF2,FOXL1,FOXM1,FOXO3,FOXO4,FOXP1, FOXP3,FOXQ1,FRAT1,FSCN1,FURIN,FZD1,GAB2,GAL,GALNT9,GAS6,GATA2,GATA5,

GATA6,GBP2,GC,GCG,GDF15,GGH,GIP,GIPC1,GLA,GLI1,GNAQ,GOLPH3,GPRC5A,GPX3
,GRB2,GRM4,GSTP1,GTSE1,HAPLN3,HDAC1,HDAC2,HDAC6,HDGF,HES1,HIF1A,HK1,
HMGA1,HMGA2,HMGB3,HNF1A,HOMER1,HOXA13,HOXA9,HOXB9,HPSE,HR,HSD3B1,
HSPA2,HTATIP2,HTRA2,HTRA3,HUWE1,HYOU1,ID2,ID3,IGF1R,IGFBP7,IL10,IL13,ILK,IMP
3,
IMPACT,ING3,IQGAP1,IQGAP2,IRX5,ITGA3,ITGA8,ITGB1,ITGB3,ITGB4,ITGBL1,JAK2,
JMJD6,JUN,KCNJ1,KDM3A,KDM5C,KDR,KEAP1,KIAA0101,KIF14,KIF18A,KIF26B,KIF2A,
KISS1,KIT,KLF17,KLF4,KLF6,KLK10,KLK3,KPNA2,KRAS,KRT7,LAMP3,LAPTM4B,LAT,LEP
,
LETM1,LIFR,LIG4,LIN28B,LMO7,LOX,LOXL2,LRG1,LYN,LZTS1,MAGEC2,MAGI1,MAML2,
MAP4K3,MAP4K4,MARCKS,MAX,MB,MBP,MCM2,MET,MGMT,MGST1,MIA,MIB1,MIF,MIP,
MITF,MMP11,MMP13,MMP16,MMP14,MMP7,MMP9,MPO,MSX2,MTA1,MTA2,MTA3,MTBP,
MTDH,MTHFD2,MTOR,MTSS1,MTUS1,MUC16,MUC2,MUC4,MVD,MVP,MYC,MYCN,MYD8
8,MYH9,MZF1,NAT1,NCK1,NCK2,NCOA2,NCOA5,NCOR1,NCOR2,NCSTN,NDRG2,NDRG3
,
NDRG4,NEDD4L,NEK2,NETO2,NEU3,NKD1,NKTR,NLK,NME1,NOB1,NOD2,NODAL,
NOTCH1,NOTCH3,NOTCH4,NOV,NPM1,NQO2,NR2F2,NR4A2,NRAS,NUCB2,NUCKS1,OA
T,OLA1,OLIG1,ORAI1,OTP,OTUB1,P4HA2,PAFAH1B1,PAK4,PARP1,PARVB,PAX3,PAX6,
PAX8,PBK,PBRM1,PBX3,PC,PCDH10,PCDH9,PCNA,PDCD4,PDCD6,PDGFRA,PEG10,PE
R1,PER2,PFKFB2,PFN2PHIP,PHLDA1,PHLPP1,PHLPP2,PIGR,PIK3CA,PIK3CB,PIK3R1,PI
P4K2B,PITX2,PIWIL2,PLA2G16,PLAGL1,PLAUR,PML,POLE,POMC,PPARG,PPM1D,PRAM
E,PRDX1,PRDX4,PRL,PROK1,PROM1,PROX1,PRSS3,PRUNE,PSCA,PTEN,PTGIS,PTGS2
,PTK7,
PTOV1,PTP4A3,RAB25,RAB27B,RAC1,RACGAP1,RAD50,RAD51,RALBP1,RALY,RAN,RA
SGRP3,RBM3,RBX1,REG4,RELA,REPS2,RET,RGS1,RGS6,RHO,RHOC,RIPK2,ROBO1,RO
CK1,ROCK2,ROR1,ROR2,RRM1,RUNX2,RUNX3,S100A11,S100A2,S100A6,S100A9,S100B
,
S100P,SATB2,SCRIB,SCUBE2,SDC1,SDC2,SDHA,SELP,SEMA3F,SERPINB2,SETDB1,
SF3B1,SFRP1,SGTA,SH2B1,SIAH2,SIPA1,SIRT1,SIX3,SIX4,SKI,SKP2,SLC19A1,SLC9A9,
SLC22A18,SLC25A1,SLC29A3,SLCO1B1,SLCO1B3,SLN,SMAD1,SMAD2,SMAD3,SMAD4,
SMAD7,SMYD2,SMYD3,SNAI2,SOCS3,SOD2,SOX10,SOX11,SOX17,SOX2,SOX4,SOX7,
SOX8,SOX9,SP1,SPAG5,SPARC,SPARCL1,SRC,SRPK1,SST,STAG2,STARD10,STARD13,
STAT1,STAT3,STAT6,STC2,STIP1,STS,STYK1,SULT1E1,SUZ12,SYK,TACC2,TACSTD2,
TAT,TBX2,TCF3,TEK,TFE3,TERT,TET1,TF,TFF3,TFPI,TGFB1,TGFBI,TGM2,TGM3,THY1,
TIMM17A,TKTL1,TLR4,TMPRSS2,TMPRSS3,TMPRSS4,TP53,TPD52,TRAF2,TRAF6,TRPM
7,TRPS1,TSG101,TSPYL5,TUBB3,TWIST1,TYMS,UBE2C,UCN,USP10,USP14,USP22,USP
9X,VAV3,VDAC1,VHL,VIP,WNT10A,WNT10B,WNT11,WNT2,WNT3A,WNT7A,WWOX,WWP
1,
XPA,XPO5,YY1,ZEB2,ZFX,ZMAT1,ZNF217

S6A Table. ROC-AUC comparisons of panMPS and other MPS versions developed by using subsets of genes based on Z_{genes} score thresholds and clump representations.

Distribution	No. of genes	No. of clumps	MSK_Prostate	Duke_Prostate	Montefiore_TNBC	MSK_Lung
*Z_{genes} score ≥ 4	33	21	0.69	0.70	0.73	0.87
Z_{genes} score ≥ 4	43	21	0.68	0.73	0.77	0.88
*Z_{genes} score ≥ 3	68	43	0.71	0.70	0.73	0.93
Z_{genes} score ≥ 3	100	43	0.70	0.72	0.74	0.94
panMPS	295	67	0.71	0.72	0.75	0.94

*Only highest Zgenes-score gene in clump

S6B Table. Linear regression model (r^2) between panMPS and other MPS versions developed by using subsets of genes based on Z_{genes} score thresholds and clump representations.

Distribution	No. of genes	No. of clumps	MSK_Prostate	Duke_Prostate	Montefiore_TNBC	MSK_Lung
* Z_{genes} score ≥ 4	33	21	0.89	0.87	0.83	0.83
Z_{genes} score ≥ 4	43	21	0.93	0.92	0.84	0.86
* Z_{genes} score ≥ 3	68	43	0.94	0.94	0.93	0.94
Z_{genes} score ≥ 3	100	43	0.97	0.97	0.94	0.96
panMPS	295	67	1	1	1	1

*Only highest Z_{genes} score gene in clump

S6C Table. Linear regression model (r2) between panMPS and other MPS versions developed by using subsets of genes based on Z_{genes} score thresholds and clump representations for CCLE.

Distribution	No. of genes	No. of clumps	Breast (n=20)	Lung Adeno (n=15)	Large Intestine (n=11)	Pancreas (n=12)	Stomach (n= 11)	Lymphoid Tissue (n=20)	Melanoma (n=17)	Lung Small Cell CA (n=22)
* Z_{genes} score ≥ 4	33	21	0.8	0.9	0.84	0.69	0.91	0.76	0.86	0.83
Z_{genes} score ≥ 4	43	21	0.85	0.92	0.9	0.7	0.96	0.73	0.89	0.91
* Z_{genes} score ≥ 3	68	43	0.92	0.96	0.98	0.79	0.94	0.88	0.96	0.95
Z_{genes} score ≥ 3	100	43	0.97	0.98	0.99	0.85	0.97	0.92	0.97	0.98

*panMPS	175	67	0.95	0.97	0.99	0.9	0.94	0.95	0.98	0.97

*Only highest Z_{genes} score gene in clump

Table S7. Studies Validating Commercially Available Molecular Biomarkers Alone or Combined with Clinical Markers for Prostate Cancer Outcome Risk Prediction. Endpoints include Adverse Pathology, Biochemical Recurrence, Metastasis Recurrence, and Metastasis-Free Survival

Test	Study Design	Outcome	Results
Oncotype Dx Prostate Genomic Prostate Score (GPS)[21]	**Cohort.** Biopsies and radical prostatectomy specimens for very low-, low-, or intermediate-risk prostate cancers (N = 431) between 1990 and 2011. **Intent.** Validation study to test the associations between GPS and biochemical recurrence (BCR), adverse pathology (AP), and metastatic recurrence (MR).	AP	AUC = 0.72 (GPS + NCCN) AUC = 0.63 (NCCN)
		BCR	Not reported
		MR	Not reported
Genomic Prostate Score (GPS)[22]	**Cohort**. Biopsies and radical prostatectomy specimens for low to intermediate clinical risk prostate cancers (N = 395) between 1997 and 2011. **Intent.** Validation study to test the association between GPS and adverse pathology (AP).	AP	AUC = 0.67 (GPS + CAPRA) AUC = 0.63 (CAPRA)
Cell Cycle Progression Score (CCP)[23]	**Cohort 1.** Radical prostatectomy specimens for low- , intermediate-, or high-risk prostate cancers (N=366) consecutive cases from 1985 to 1995. **Cohort 2.** Transurethral resection of prostate (TURPs) for low-, intermediate-, and high-risk cancers (N=337) randomly selected from 1990 to 1996. **Intent.** Validation study to test the association between CCP score and BCR and prostate cancer-specific mortality.	BCR (RP cohort)	AUC = 0.842 (clinical scores+ CCP) AUC = 0.825 (clinical scores)
		Death from prostate cancer (TURP cohort)	AUC = 0.878 (clinical scores + CCP) AUC = 0.806 (clinical scores)
Genomic Classifier (GC)[24]	**Cohort.** Radical prostatectomy samples from 85 clinically high-risk patients who developed BCR +/- metastases after RP.	MFS	AUC = 0.82 (GC scores) AUC = 0.64 (GS) AUC = 0.69 (PSAdT) AUC = 0.52 (ttBCR)

	**Intent.** Validation study to compare GC to Gleason score (GS), PSA doubling time (PSAdT), time to BCR (ttBCR), Stephenson nomogram and CAPRA-S for predicting metastasis-free survival (MFS), 5-year metastatic risk after biochemical recurrence.		**Comment.** In multivariable modeling with clinicopathologic variables, GC score was the only significant predictor of metastasis.
Genomic Classifier (GC) [25]	**Cohort**. Nested case-control, high-risk, radical prostatectomy samples from 2000-2006 (N=185).<br><br>**Intent.** Validation study to test association between GC and CAPRA-S for predicting risk of prostate cancer specific mortality.	Prostate cancer specific mortality	AUC = 0.75 (CAPRA-S)<br><br>AUC = 0.78 (GC)<br><br>**Comment.** Combining both did not improve AUC
Genomic Classifier (GC) [26]	**Cohort.** Nested case-control, radical prostatectomy samples from 1987-2001, including training set (N=359) and validation set (N=186).<br><br>**Intent.** Validation study to tested association between GC and clinical classifiers, including pathologic Gleason score, preoperative PSA, surgical margins, seminal vesicle involvement, extracapsular extension and lymph node extension for predicting metastasis-free survival (MFS), 5-year metastatic risk after biochemical recurrence.	MFS	AUC = 0.74 (GC + clinical classifier)<br><br>AUC = 0.75 (GC)<br><br>AUC = 0.69 (clinical classifier)
Genomic Classifier (GC) [27]	**Cohort.** Radical prostatectomy samples from 219 clinically high-risk patients who +/- developed biochemical recurrence (BCR) +/- metastases after RP.<br><br>**Intent.** Validation study to test association between GC and clinical classifiers - GPSM (Gleason score, preoperative PSA, seminal vesicle involvement, surgical margins) and Stephenson nomogram for predicting metastasis-free survival (MFS), 5-year metastatic risk after biochemical recurrence.	MFS	AUC = 0.79 (GC scores)<br><br>AUC = 0.46 - 0.64 (clinical variables)<br><br>**Comment.** The genomic classifier was the predominant predictor of metastasis on multivariable analysis.
Genomic Classifier (GC) [28]	**Cohort**. Cohort study of radical prostatectomy specimens for high-risk (pT3 or positive margin) prostate cancers following radiation therapy (RT) (N=139).	BCR	AUC = 0.78 (GC + Stephenson nomogram)<br>AUC = 0.75 (GC)<br>AUC = 0.70 (Stephenson nomogram)<br>AUC = 0.67 (CAPRA-S)

	**Intent.** Comparison between GC and clinical nomogram (Stephenson) for predicting BCR and metastasis.	Metastasis	AUC = 0.80 (GC + Stephenson nomogram)<br>AUC = 0.78 AUC (GC)<br>AUC = 0.70 (Stephenson nomogram)<br>AUC = 0.65 (CAPRA-S)
			**Comment.** Neither clinical nomogram was significant for predicting metastasis. (For both the CI included 0.50.)

AP = adverse pathology; AUC = area under the curve; BCR = biochemical recurrence; CAPRA = Cancer of the Prostate Risk Assessment Score; CCP = Cell Cycle Progression Score; GC = Genomic Classifier; GPS = Genomic Prostate Score; GPSM = Gleason score, preoperative PSA, seminal vesicle involvement, surgical margins; GS = Gleason score; MFS = metastasis-free survival; MR = metastatic recurrence; NCCN = National Comprehensive Cancer Network; PSA = prostate specific antigen; PSAdT = PSA doubling time; RP = radical prostatectomy; RT = radiation therapy; ttBCR = time to biochemical recurrence; TURP = transurethral resection of prostate.

S8A Table. Clinical and histological characteristics of samples used to validate the panMPS model for metastatic outcome for prostate cancer

**Cohort**	**MSK Prostate CA**		**Duke Prostate CA**		**P**
**Outcome**	**mPT**	**iPT**	**mPT**	**iPT**	
n	25	260	37	39	
**Age**					
Mean	58.93	58.15	64.19	61.82	0.5
Median	59.47	58.13	64	62	
Standard deviation	7.23	6.82	6.48	8.41	
Range	46-71	37-75	47-77	46-77	
**Clinical stage**					
T1C	10 (3.51%)	146 (51.22%)	18 (23.68%)	24 (31.57%)	7.56×10^{-8}
T2	11 (3.86%)	106 (37.19%)	9 (11.84%)	4 (5.26%)	3.86×10^{-6}
T3	4 (1.40%)	8 (2.81%)	0 (0%)	0 (0%)	
**Path stage**					
T2	7 (2.46%)	156 (54.74%)	6 (7.89%)	9 (11.84%)	1.53×10^{-4}
T3	13 (4.56%)	92 (32.28%)	24 (31.57%)	26 (34.21%)	3.85×10^{-6}
T4	5 (1.75%)	12 (4.21%)	7 (9.21%)	4 (5.26%)	0.12
**Biopsy Gleason score**					
3	0 (0%)	0 (0%)	0 (0%)	1 (1.31%)	
4	0 (0%)	0 (0%)	1 (1.31%)	0 (0%)	
5	0 (0%)	2 (0.70%)	0 (0%)	4 (5.26%)	
6	7 (2.46%)	142 (49.82%)	10 (13.51%)	18 (23.68%)	1.91×10^{-5}
7	12 (4.21%)	92 (32.28%)	14 (18.42%)	12 (15.78%)	1.19×10^{-5}

8	5 (1.75%)	15 (5.26%)	5 (6.57%)	0 (0%)	0.01
9	0 (0%)	9 (3.16%)	3 (3.94%)	4 (5.26%)	0.06
10	0 (0%)	0 (0%)	1 (1.31%)	0 (0%)	
**Path Gleason score**					
6	1 (0.35%)	68 (23.86%)	2 (2.63%)	2 (2.63%)	0.01
7	8 (2.81%)	173 (60.70%)	20 (26.31%)	29 (38.16%)	9.27×10^{-10}
8	6 (2.11%)	10 (3.51%)	2 (2.63%)	3 (3.95%)	
9	19 (6.66%)	8 (2.81%)	12 (15.785)	5 (6.58%)	
10	0 (0%)	0 (0%)	1 (1.31%)	0 (0%)	
**Preop PSA (ng/mL)**					
Median	8.49	5.6	7.5	7.3	
< 4	4 (1.40%)	46 (16.14%)	4 (5.26%)	4 (5.26%)	
4–10	10 (3.51%)	163 (57.19%)	16 (21.05%)	27 (35.52%)	
>10	11 (3.86%)	50 (17.54%)	17 (22.36%)	8 (10.52%)	

P-values were determined by Wilcoxon Rank Sum Test and Fisher's Exact Test respectively for continuous and categorical variables for inter cohort significance.

S8B Table. Clinical and histological characteristics of samples used to validate the panMPS model for metastasis outcome for TNBC

Cohort	Montefiore TNBC	
**Outcome**	**mBC**	**iBC**
n	28	13
**Age**		
Mean	58.3	53
Median	61.5	49
Standard deviation	11.57	11.7
Range	35-82	34-74
**TNM Stage**		
T1	4 (9.75%)	5 (12.19%)
T2	7 (17.07%)	5 (12.19%)
T3	4 (9.75%)	0 (0%)
T4	2 (4.88%)	0 (0%)

S8C Table. Clinical and histological characteristics of samples used to validate the panMPS model for metastasis outcome for lung adenocarcinoma

Cohort	MSK Lung Adeno CA	
Outcome	mLA	iLA
n	23	10
**Sex**		
Male	11 (26.83%)	4 (9.76%)
Female	12 (29.27%)	6 (14.63%)
**TNM Stage**		
1B	6 (14.63%)	0 (0%)
2	7 (17.07%)	1 (2.44%)
3	10 (24.39%)	5 (12.20%)
4	2 (4.88%)	4 (9.76%)

S9 Table: Description of Metabric and TCGA cohorts

Cohort Name	Sample Size
Metabric Study for Breast invasive carcinoma	2173
TCGA provisional study for Breast invasive carcinoma	1080
TCGA provisional study for Lung adenocarcinoma	516
TCGA provisional study for Prostate adenocarcinoma	492

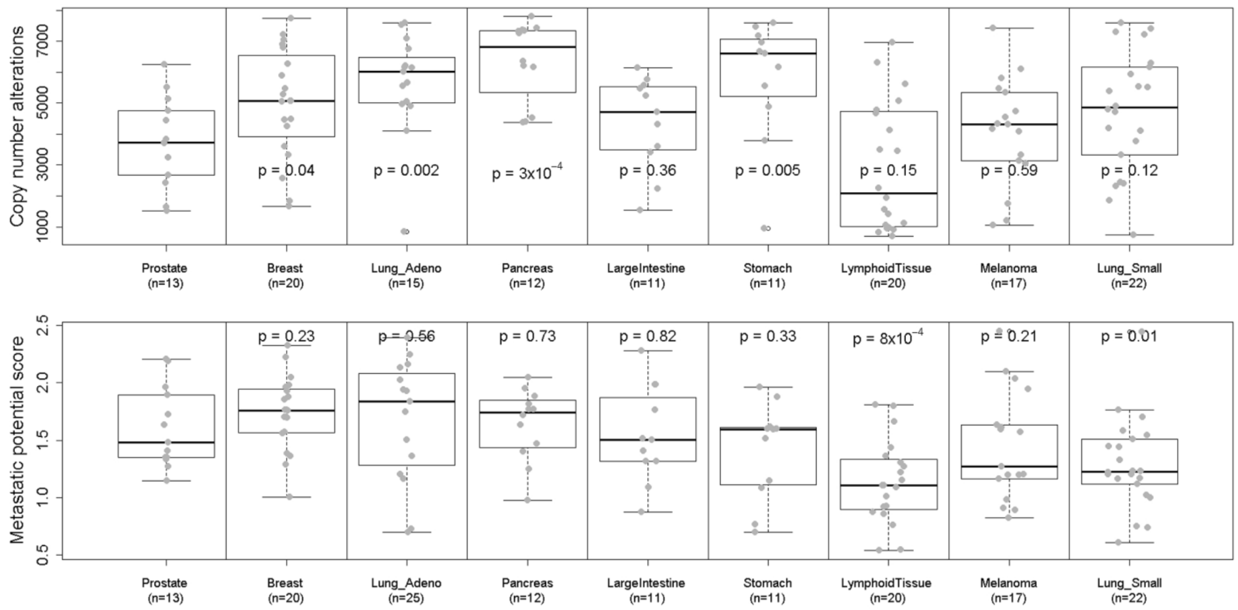

S1 Figure: Genomic instability in CCLE cell lines of metastatic origin is elevated compared to clinical prostate cancer metastases, whereas MPS was similar for many cell types, especially those of epithelial origin. (Top panels) Boxplots of total gene copy number alterations by CCLE cell type and clinical prostate cancer metastases. (Bottom panels) Boxplots of MPS for each CCLE cell type and clinical prostate cancer metastases. P-values show pairwise comparisons between individual CCLE cell type versus prostate cancer metastases using the Mann-Whitney test.

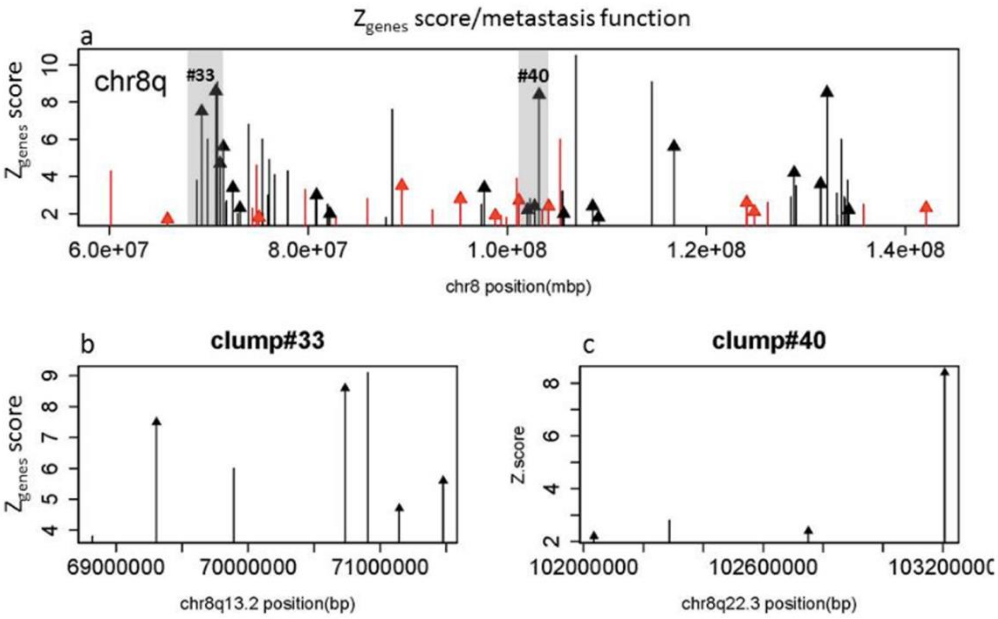

S2 Figure: Chromosome 8q comprises 75 genes predictive of metastatic potential, including genes that occur in clumps (top panel). Each bar represents a gene location on the chromosome (X-axis, base pair numbers) whereas the height of the bar denotes a Z_{genes} score (Y-axis) that predicts the metastatic potential of a primary prostate cancer. Arrows on top of some of the bars indicate that the gene has been validated in prior metastasis studies as a biomarker or to have metastatic function. Red colored bars/arrows indicate a singleton gene or one member clumps. Clump #33 (six-gene segment) and clump #40 (four-gene segment) are highlighted in the top panel and zoomed in the bottom panels.

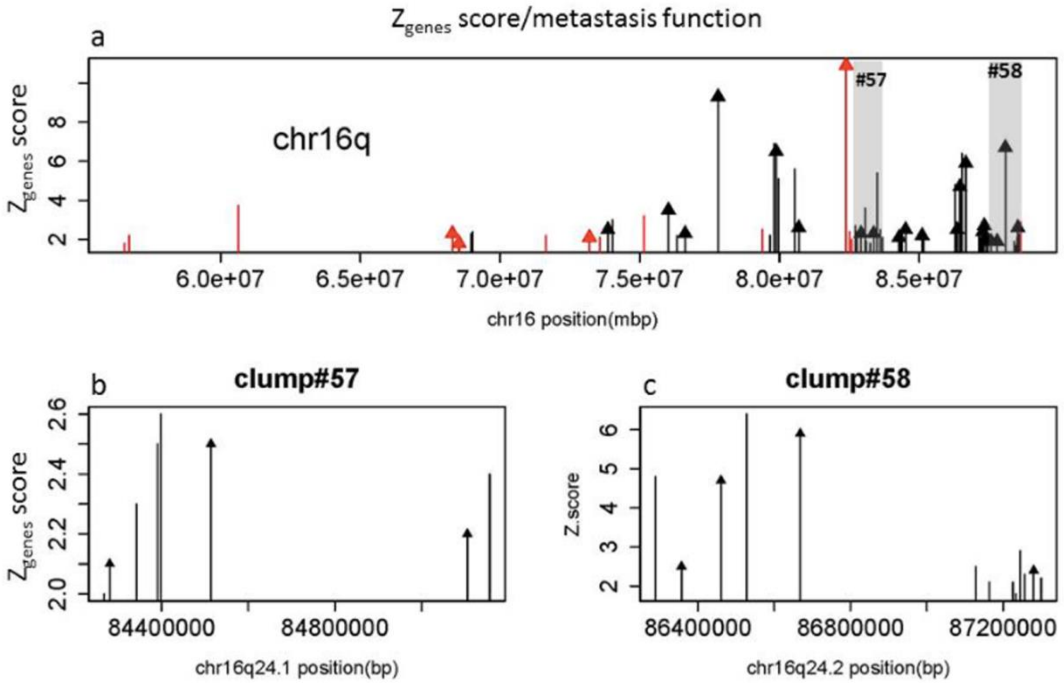

S3 Fig: Chromosome 16q comprises 74 genes predictive of metastatic potential, including genes that occur in clumps (top panel). Each bar represents a gene location on the chromosome (X-axis, base pair numbers) while the height of the bar denotes a Z_{genes} score (Y-axis) that measures the the metastatic potential of a primary prostate cancer tumor. Arrows on top of some of the bars indicate that the gene has been validated in prior metastasis studies as a biomarker or to have metastatic function. Red colored bars/arrows indicate a singleton gene or one member clump. Clump #57 (8-gene segment) and clump #58 (13-gene segment) are highlighted in the top panel and zoomed in the bottom panels.
